# DRG-LiteStar-YOLO: Reliability-Guided Pseudo-Depth Fusion for Dairy Goat Detection in Complex Barns

**DOI:** 10.3390/ani16182840

**Published:** 2026-09-09

**Authors:** Yongliang Zhang, Keyuan Wang, Yue Yang, Nan Geng

**Affiliations:** 1 College of Information Engineering, Northwest A&F University, Xianyang 712100, China; yuc_neo@nwafu.edu.cn (Y.Z.);; 2 Key Laboratory of Agricultural Internet of Things, Ministry of Agriculture and Rural Affairs, Northwest A&F University, Xianyang 712100, China; 3 Shaanxi Key Laboratory of Agricultural Information Perception and Intelligent Service, Northwest A&F University, Xianyang 712100, China

**Keywords:** dairy goat detection, precision livestock monitoring, pseudo-depth map, RGB–depth fusion, YOLO, depth reliability

## Abstract

Reliable dairy goat detection in barns is challenging because uneven illumination, railings, and animal overlap weaken RGB boundaries. We developed DRG-LiteStar-YOLO, a lightweight RGB-camera-compatible detector that integrates monocular pseudo-depth with RGB features without requiring a dedicated depth sensor. DA3-Small was used to generate relative pseudo-depth maps, which were fused with RGB features through reliability-guided fusion and boundary-enhanced multi-scale aggregation. The dataset comprised 1521 images of 134 lactating Saanen goats and 13,985 annotated instances. On the held-out test set, DRG-LiteStar-YOLO achieved a precision of 0.961, a recall of 0.941, an mAP@0.5 of 0.978, and an mAP@0.5:0.95 of 0.735. Compared with RGB-only LiteStar-YOLO, the proposed method improved mAP@0.5 and mAP@0.5:0.95 by 1.6 and 3.8 percentage points, respectively. The five-seed cumulative ablation further showed that the full configuration achieved 0.734±0.002 mAP@0.5:0.95 compared with 0.697±0.004 for RGB-only LiteStar-YOLO. The detector contains 4.20 M parameters and 12.60 GFLOPs and achieves 86.2 FPS on an RTX 4090 with precomputed pseudo-depth maps. These results demonstrate that reliability-guided pseudo-depth fusion improves goat localization under complex barn conditions while retaining a compact detector design. DRG-LiteStar-YOLO provides an effective perception framework for RGB-camera-based dairy goat monitoring and supports future counting, tracking, and behavior-analysis applications.

## Simple Summary

Farm cameras can help workers monitor dairy goats without repeatedly entering pens, but automatic detection becomes difficult when animals overlap, railings cross their bodies, or lighting changes sharply. We developed a computer vision model that combines ordinary color images with depth-like information estimated from the same images. Before feature fusion, the model estimates a task-specific reliability weight for the pseudo-depth information and uses depth-boundary cues to help separate neighboring goats. We evaluated the method using 1521 original images collected from 134 lactating Saanen goats in a university barn. Compared with a lightweight RGB-only detector, the proposed method improved mAP@0.5 and mAP@0.5:0.95 by 1.6 and 3.8 percentage points, respectively. In the cumulative ablation, five prespecified seeds produced a full-model mAP@0.5:0.95 of 0.734±0.002 compared with 0.697±0.004 for RGB-only LiteStar-YOLO. With pseudo-depth maps prepared offline, the detector processed 86.2 images per second; this speed excludes pseudo-depth generation. These results demonstrate that pseudo-depth can provide useful geometric information for improving goat localization without requiring a dedicated depth camera. The proposed framework offers a practical perception module for within-farm goat monitoring and provides a foundation for subsequent counting, tracking, and behavior-analysis systems. The present evaluation focused on single-frame detection in one university barn.

## 1. Introduction

Precision livestock farming (PLF) uses continuous sensing to support decisions about animal health, welfare, behavior, and production [1,2]. Vision-based systems provide detection, segmentation, pose estimation, tracking, and behavior recognition across several livestock species [3,4]. Object detection has a narrower role than these downstream tasks: it localizes animals in individual frames but does not itself quantify behavior, infer emotional state, or assess health. Stable frame-level localization is nevertheless an upstream perception requirement for many detection–tracking and behavior-analysis pipelines, where animal trajectories or localized image regions are subsequently associated over time [5,6].

For goats, prior studies have addressed surveillance-video detection, counting, tracking, segmentation, individual recognition, and behavior recognition [5,7,8,9,10,11,12]. Recent work has extended lightweight models to multi-behavior recognition in dairy goats and detection–tracking pipelines in dairy cattle [6,12]. These studies show the value of accurate localization, but their datasets are usually farm- and task-specific. Limited breed, camera, and environmental coverage can cause a detector to learn scene-specific backgrounds or viewpoints, weakening transfer to other goat herds and commercial barns [13].

One-stage YOLO detectors are widely used when inference speed matters [14,15,16]. Compact backbones, attention, and feature pyramids can preserve appearance information across channels and scales [17,18,19], but they cannot reconstruct geometry that is absent from weak RGB texture. True depth sensors provide calibrated range information and can remain informative when color contrast is poor. RGB–infrared livestock detectors similarly show that a second measured modality can help under low light and occlusion [20]. Such systems require additional hardware, calibration, synchronization, and maintenance, which limits direct deployment in barns equipped only with RGB cameras.

Monocular depth estimation offers a software-only alternative. Single-image prediction was established with early deep networks [21] and later improved through mixed-dataset training, transformer-based dense prediction, and large-scale pseudo-labeling [22,23,24,25,26]. For barn cameras, however, these models provide relative pseudo-depth rather than calibrated physical distance. Shadows, overexposure, reflective surfaces, railings, occlusion, and unfamiliar viewpoints can distort local geometry. Pseudo-depth therefore reduces sensor requirements at the cost of accuracy, robustness, and additional preprocessing computation.

The unresolved problem is how to use this imperfect geometry without allowing local pseudo-depth errors to dominate RGB features. Direct concatenation treats all depth responses as equally informative. Confidence-aware RGB-D methods instead calibrate or attenuate low-quality depth during fusion [27,28,29,30]. Boundary-aware networks preserve contours that ordinary feature pyramids can lose during repeated resizing [31,32]. These ideas have rarely been combined under the sensor and efficiency constraints of dairy goat detection in crowded barns.

We therefore developed DRG-LiteStar-YOLO for single-frame goat localization. The central design choice is to treat monocular pseudo-depth as uncertain auxiliary evidence: an agreement-derived gate attenuates inconsistent depth features, and explicit depth boundaries are preserved during cross-modal and multi-scale fusion. The study tests this design against RGB-only detectors and representative fusion strategies while retaining compatibility with conventional RGB cameras. It does not evaluate tracking, behavior, emotion, or health outcomes. The work makes four bounded contributions:

- It formulates monocular geometry as uncertain auxiliary evidence for dairy goat detection, avoiding the need for a dedicated depth sensor.
- It combines agreement-based depth gating with explicit boundary preservation at the fusion and feature-pyramid stages, rather than applying unweighted RGB–depth concatenation.
- It evaluates the integrated design through detector comparisons, cumulative ablations, and representative fusion configurations on an unaugmented held-out test partition.
- It quantifies detector-only accuracy and efficiency while explicitly separating these results from unmeasured depth-generation latency and downstream animal- monitoring tasks.

## 2. Related Work

### 2.1. Computer Vision for Precision Livestock Monitoring

Livestock vision research covers detection, tracking, segmentation, identification, and behavior recognition [3,4]. RGB detection has been combined with multi-object tracking in cattle [33], while recent lightweight models address dairy goat behavior and dairy cow detection–tracking pipelines [6,12]. These systems differ in animal species, labels, camera placement, and evaluation protocol. Public datasets also vary in scene diversity, contextual metadata, and annotation rules, so numerical results cannot be treated as direct cross-study rankings [13].

Goat studies have used Faster R-CNN for surveillance-video detection [7] and convolutional models for group behavior and facial recognition [8,34]. Other work has addressed lightweight tracking, sheep–goat face classification, counting, segmentation, individual recognition, and multiple behaviors [5,9,10,11,12,35]. The present task stops at frame-level detection. Its technical focus is the localization failure caused by railings, overlapping bodies, and weak coat–background contrast, rather than temporal identity or behavioral classification.

Compact YOLO variants reduce detector cost [6,12], and lightweight attention can refine spatial responses with modest model overhead [17]. RGB–infrared fusion is a recent livestock-specific example of measured multimodal sensing under low light and occlusion [20]. RGB-only systems are simpler but lack explicit range cues; sensor-based RGB-D or RGB–infrared systems add stronger measurements but require extra hardware; pseudo-depth systems preserve the RGB camera infrastructure but introduce estimation error and preprocessing latency. DRG-LiteStar-YOLO occupies the third setting.

### 2.2. Lightweight YOLO-Based Object Detection

Two-stage detectors first generate proposals and then classify them. Faster R-CNN is a representative example [36], but its computational cost can hinder continuous barn monitoring. One-stage models predict classes and boxes directly from dense feature maps. SSD and YOLO therefore offer a more practical speed–accuracy balance [14,37]. Later YOLO variants improved feature extraction, training, and real-time performance [15,16].

A barn detector may run continuously on a local server or edge device, so efficiency matters. MobileNet reduced computation by separating spatial convolution from channel mixing [38]. This operation now appears in many compact detectors. Excessive compression, however, removes details that are already faint in low-light barn images. White coats, railings, and textured floors make that trade-off particularly sharp.

Livestock detectors commonly combine efficient backbones with attention or multi-scale aggregation [6,12]. Our design follows this efficiency constraint but assigns different roles to the two inputs. The RGB branch handles texture under uneven light. The depth branch and boundary-aware neck supply geometry that the compact RGB backbone may miss.

### 2.3. Pseudo-Depth Estimation and RGB–Depth Fusion

Depth separates foreground objects through relative distance and discontinuities in scene geometry. Conventional RGB-D systems obtain it from depth cameras, stereo rigs, or LiDAR. These sensors yield explicit measurements, but they also change installation, calibration, synchronization, and maintenance requirements. A monocular alternative preserves the existing RGB camera infrastructure, although its output is estimated rather than measured.

Monocular models infer relative depth from one RGB image. Mixed-dataset training improved transfer to unseen scenes [22], while dense prediction transformers strengthened global consistency [23]. Depth Anything, Depth Anything V2, and DA3 expanded large-scale visual geometry learning [24,25,26]. They make offline pseudo-depth generation feasible for standard barn footage, but their predictions are not equivalent to calibrated barn-specific depth and may shift under unusual illumination, severe occlusion, motion blur, reflective surfaces, or highly textured structures.

Estimated depth can fail locally, particularly around shadows, occlusions, saturated regions, and fine railings. Most relevant confidence-aware evidence comes from RGB-D salient object detection rather than livestock detection. That literature includes cooperative interaction [27], depth calibration [28], depth-sensitive attention [29], and fusion designed for low-quality or misaligned depth [30]. DRGF follows the general principle of attenuating inconsistent depth responses, but its learned gate is not supervised by ground-truth depth and should not be interpreted as a calibrated uncertainty estimate.

### 2.4. Multi-Scale Feature Fusion and Boundary-Aware Detection

Feature pyramids address scale changes caused by perspective and animal movement. FPN combines high-level semantics with finer spatial features through a top-down path and lateral links [18]. BiFPN adds learnable weights and a bottom-up return path [19]. Both structures aggregate features across scales, but neither explicitly retains an auxiliary geometric boundary after repeated resizing.

Boundary research provides a possible remedy. Hierarchical convolutional features improve contour extraction across scales [31], while shape streams process boundaries explicitly around thin or ambiguous structures [32]. Crowded goat scenes contain related ambiguity where railings intersect bodies and adjacent goats overlap. DBE-BiFPN therefore introduces the same pseudo-depth boundary cue at P3, P4, and P5 so that fine spatial detail and coarser object-scale context can both access it.

Existing work separately addresses efficient livestock detection, monocular depth, confidence-aware RGB-D fusion, and boundary-aware prediction. The novelty claimed here is their task-specific integration: an RGB-compatible dairy goat detector uses cross-modal agreement to gate estimated depth and retains its boundary cue during both attention and multi-scale aggregation. This is an integrated architecture contribution rather than a claim that pseudo-depth, attention, or BiFPN is individually new.

## 3. Materials and Methods

### 3.1. Study Site and Data Acquisition

Images were collected at the Animal Husbandry Teaching and Experimental Base of Northwest A&F University (NWAFU). The farm is in Yangling District, Xianyang, Shaanxi Province, China (approximately 108.08° E, 34.27° N). During collection, it housed 134 lactating Saanen goats aged 2–5 years.

Video was acquired with fixed TP-IPC44CL-V2 cameras manufactured by TP-Link Technologies Co., Ltd., Shenzhen, China, equipped with a 1/3-inch CMOS sensor and a 4-mm fixed-focus lens. The recordings were captured at 2560 × 1440 pixels and encoded using H.265/HEVC. Each camera was mounted approximately 2.5 m above the floor with a downward viewing angle of approximately 30°. Acquisition was conducted on 15 collection days between 15 March and 1 April 2026, with approximately 24 h of recording on each collection day, producing 248 source video files.

A video-sampling script implemented in Python 3.11.9 and OpenCV 4.13.0.92 decoded the H.265 recordings, sampled frames at a target rate of 10 f/s, and exported them in PNG format to form the initial image library. Fixed-interval sampling retained temporal changes in individual posture, group distribution, and illumination, but adjacent frames still contained substantial visual redundancy. We therefore applied a two-stage automatic de-duplication and manual quality-control procedure. First, a 64-bit perceptual hash (pHash) was calculated for each sampled frame. Frames from each source video were processed chronologically: a frame was assigned to the current near-duplicate group when its Hamming distance from the first frame of that group was no greater than 6; otherwise, the current group was closed and a new group was started. For every group, sharpness was measured on the original-resolution grayscale frames as the variance of a 3×3 Laplacian response computed in 64-bit floating-point precision. The frame with the largest Laplacian variance was retained as the group representative; if this maximum was below 100, the entire group was rejected as globally blurred. Second, manual inspection removed frames without a complete annotatable target, with substantial local truncation, inseparable severe overlap, global blur, or extreme illumination that made body contours unrecognizable. Ordinary occlusion and overlap were retained when individual goats remained annotatable. This procedure reduced near-duplicate, blurred, extremely exposed, and low-information images while preserving samples suitable for consistent bounding-box annotation. The final 1521 annotated source images therefore did not constitute uninterrupted frame sequences. Source-video identifiers were retained and used to prevent any one video from contributing images to both the training and test subsets.

**Figure 1:**
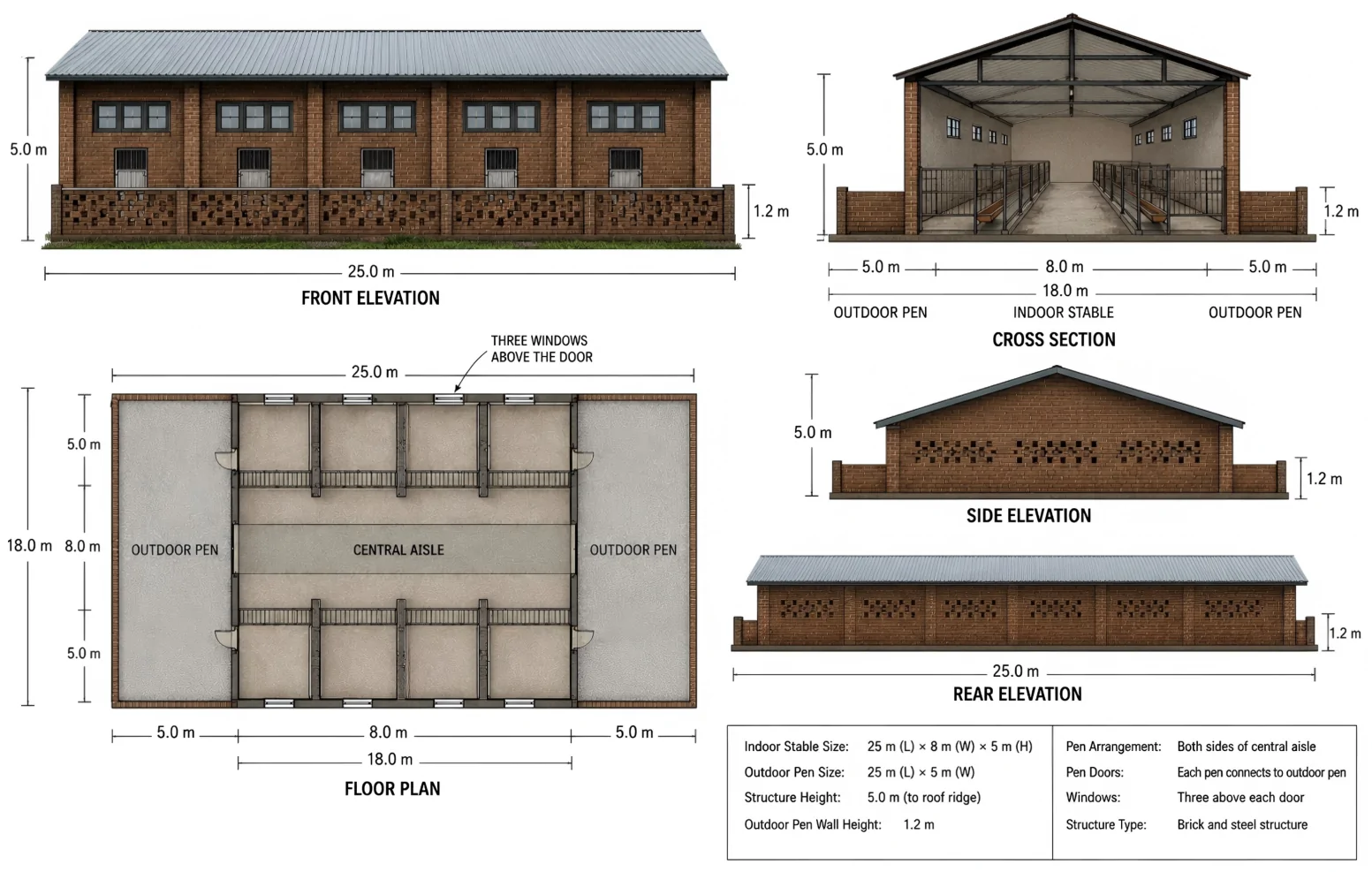
Barn dimensions and structural views used for dairy goat image acquisition.

Figure 1 shows the barn layout used for image acquisition.The main characteristics of the study site and data-acquisition environment are summarized in Table 1. The indoor stable measured approximately 25.0 m by 8.0 m and was 5.0 m high. A central aisle separated pens on either side. Each indoor pen opened into an outdoor area measuring about 25.0 m by 5.0 m. Across the stable and both outdoor pens, the structure was approximately 18.0 m wide. The outdoor walls were 1.2 m high. Brick walls and a steel roof enclosed the main stable.

Conditions varied substantially within the barn. Light entering through doors, windows, and side openings produced dark regions beside locally overexposed areas. Metal railings and feeding equipment often crossed the animals in the image plane. The goats stood, lay down, bent toward the floor, or overlapped in groups. Consequently, many frames contained incomplete contours and weak separation between neighboring bodies.

From the initial frame library, 1521 source RGB images were retained, and every visible goat received a bounding-box annotation. The source corpus contained 13,985 goat instances. The public augmentation archive retains each source image together with seven photometric variants: Gaussian blur, Gaussian noise, grayscale conversion, salt-and-pepper noise, histogram equalization, random saturation adjustment, and random contrast adjustment. These operations preserved image geometry, so the original bounding boxes were transferred without coordinate changes. The complete release archive therefore contains 12,168 image files and 111,880 annotations. This archive is not the experimental split: only variants derived from training sources were used for model fitting, producing 8520 training images and 8976 images across training, validation, and testing (Section 4.1). Together with pretrained initialization and 13,985 annotated goat instances, the 1521 independent source images permit a controlled within-farm comparison in which all detectors are trained and evaluated under the same protocol. They should not, however, be interpreted as sufficient evidence for biological, cross-camera, cross-breed, or cross-farm generalization, because augmentation does not add new animals, cameras, breeds, or barn environments.

We used the official model identifier depth-anything/DA3-SMALL to generate one pseudo-depth map for each RGB image [26]. In the official model zoo, DA3-Small is the smallest checkpoint in the main any-view series. It uses a plain transformer with a unified depth-ray representation and supports relative-depth prediction from a single RGB image. We retained only this relative-depth output for detection; camera-pose and other native DA3 outputs were not used. DA3-Small was selected to limit the offline preprocessing burden while retaining the main series’ relative-depth capability. This was a pragmatic efficiency choice rather than the result of a barn-specific comparison: we did not compare DA3 variants or alternative monocular estimators. Depth estimation was performed offline, the DA3 weights were frozen, and no depth camera was used. DA3 generation time was not recorded during preprocessing. Therefore, all latency and throughput values reported in this study describe detector-only inference with precomputed pseudo-depth maps and should not be interpreted as end-to-end RGB-to-detection performance. The complete online latency, including pseudo-depth generation and the associated preprocessing and data-transfer overhead, remains to be measured on the target deployment hardware.

### 3.2. Overall Architecture of DRG-LiteStar-YOLO

**Figure 2:**
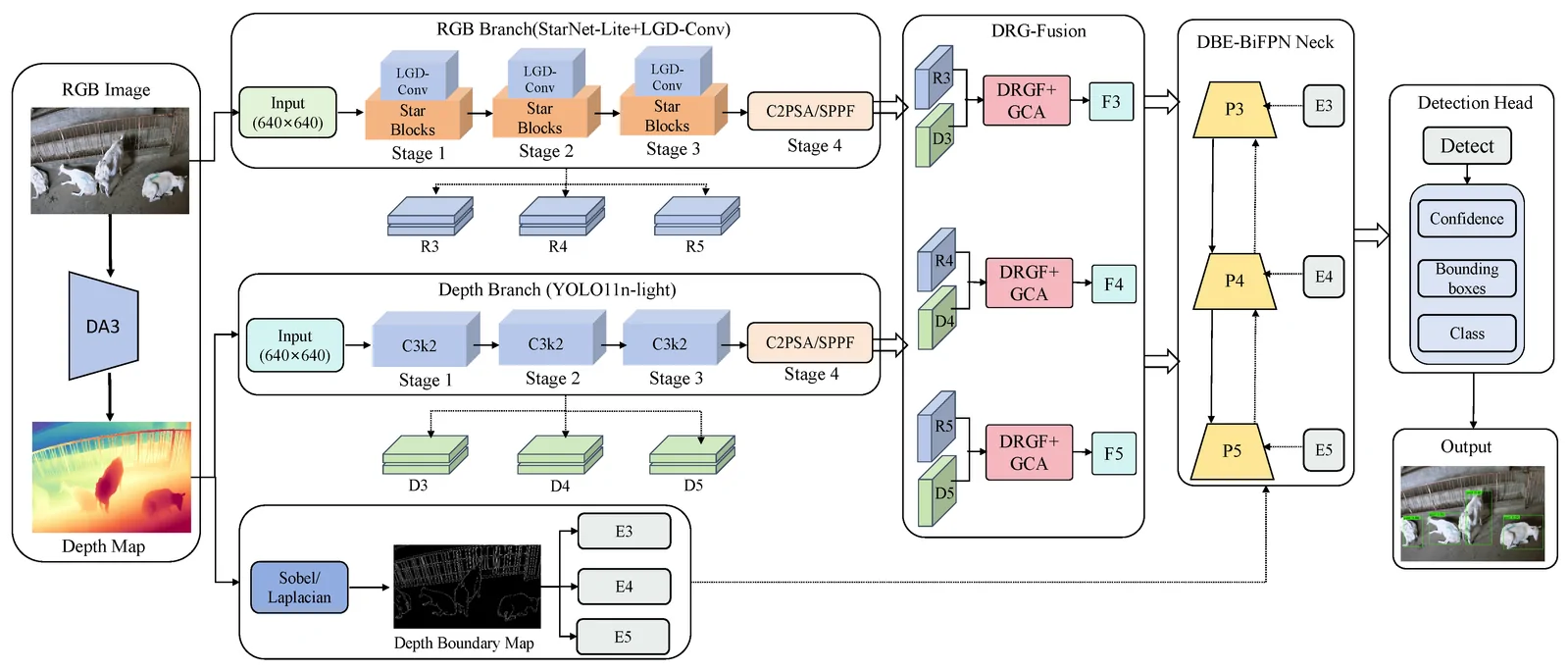
Overall architecture of the proposed DRG-LiteStar-YOLO. DA3 generates a pseudo-depth map from the RGB image. Separate branches encode appearance and geometry. DRGF weights the depth response, GCA refines cross-modal features, and DBE-BiFPN aggregates boundary-aware pyramid features.

DRG-LiteStar-YOLO has two feature streams and a shared detection neck (Figure 2). DA3 produces the auxiliary depth map before detector inference. An illumination-adaptive branch processes RGB appearance, while a smaller branch extracts geometric features. DRGF and GCA merge the streams. DBE-BiFPN then aggregates the fused features, and a decoupled YOLO head predicts classes and boxes.

For an RGB image $I^{rgb}$, monocular estimation first gives the pseudo-depth map $I^{d}$. The two inputs pass through separate branches. Their outputs at three pyramid levels are

(1) $$\left\{R_{3},R_{4},R_{5}\right\}=\Phi_{rgb}\left(I^{rgb}\right),$$

(2) $$\left\{D_{3},D_{4},D_{5}\right\}=\Phi_{d}\left(I^{d}\right),$$

where $\Phi_{rgb}\left(\cdot \right)$ and $\Phi_{d}\left(\cdot \right)$ are the RGB and depth branches. Levels 3, 4, and 5 have strides of 8, 16, and 32.

A parallel boundary path applies Sobel filtering to $I^{d}$ and resizes the result for each pyramid level:

(3) $$\left\{E_{3},E_{4},E_{5}\right\}=R\left(S(I^{d})\right).$$

Here, S(·) denotes Sobel boundary extraction and R(·) denotes scale-specific resizing. DRGF and GCA then process each RGB-depth feature pair:

(4) $$F_{l}=F_{DRG}\left(R_{l},D_{l},E_{l}\right),\;l\in \left\{3,4,5\right\}.$$

DBE-BiFPN aggregates $\left\{F_{3},F_{4},F_{5}\right\}$ together with $\left\{E_{3},E_{4},E_{5}\right\}$. The detection head uses the resulting features for classification and box regression.

### 3.3. DA3 Pseudo-Depth Generation and Boundary Extraction

The method derives depth-like information from each RGB frame rather than from an additional sensor. DA3-Small belongs to the official main any-view series and uses a plain transformer with a unified depth-ray prediction target [26]. In single-image use, it predicts relative scene layout and local discontinuities that may separate goats from the background. Its compact position within the DA3 family motivated its use in an efficiency-oriented pipeline; this study did not establish that it is more accurate than larger DA3 checkpoints or other monocular estimators on barn images. The detector receives only the normalized single-channel relative-depth prediction. Color maps appear only in the figures.

Given an RGB image $I^{rgb}$, the pseudo-depth map is obtained as

(5) $$I^{d}=D_{DA3}\left(I^{rgb}\right),$$

where $D_{DA3}\left(\cdot \right)$ is the frozen DA3-Small model used offline. Only its relative-depth field was retained; no camera-pose output was passed to the detector. The DA3 implementation also exposes a model-internal confidence output associated with its depth prediction [26]. This confidence field was not supplied to the detector in the present study. Instead, DRGF was designed to estimate a task-specific fusion weight from the agreement and disagreement between RGB and pseudo-depth features. This choice separates the downstream fusion mechanism from the depth estimator’s own confidence prediction and avoids assuming that confidence learned for monocular geometry estimation is directly calibrated for goat-detection error. Consequently, the DRGF output should not be interpreted as DA3 confidence or as a probability that the predicted depth is physically correct. Because both modalities originate from the same image, they are spatially aligned at preprocessing time.

A Sobel operator converts local depth changes into horizontal and vertical gradients:

(6) $$G_{x}=K_{x}*I^{d},\;G_{y}=K_{y}*I^{d},$$

where $K_{x}$ and $K_{y}$ are the Sobel kernels and ∗ denotes convolution. Their magnitude provides the boundary response:

(7) $$E=\sqrt{G_{x}^{2}+G_{y}^{2}}.$$

The boundary map *E* is resized to three feature pyramid scales:

(8) $$E_{l}=R_{l}\left(E\right),\;l\in \left\{3,4,5\right\}.$$

**Figure 3:**
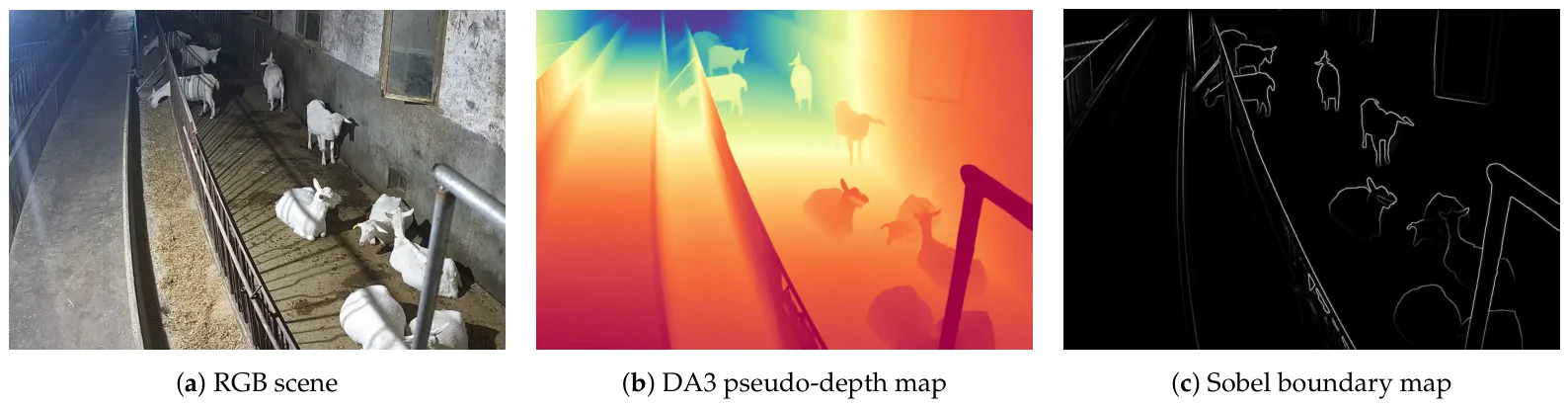
Pseudo-depth and boundary extraction in a representative barn scene: (**a**) RGB image, (**b**) DA3 pseudo-depth prediction, and (**c**) Sobel boundary map.

Here, $R_{l}\left(\cdot \right)$ resizes *E* to pyramid level *l*. The resulting $E_{3}$, $E_{4}$, and $E_{5}$ correspond to P3, P4, and P5. Figure 3 shows representative inputs and boundary responses.

### 3.4. Illumination-Adaptive RGB Branch

The RGB stream must retain texture across abrupt lighting changes. Dark pens, bright windows, and cast shadows can all weaken appearance features in the same frame. We therefore use a compact backbone derived from StarNet [39], which we term StarNet-Lite, together with LGD-Conv for illumination-conditioned channel adjustment. LGD-Conv was selected because its local path preserves fine texture, its pooled paths incorporate broader illumination context, and its depthwise separable operations keep the adaptation lightweight. Unlike a generic channel–spatial attention block such as CBAM [17], LGD-Conv explicitly combines local, pooled-context, and low-frequency residual responses before channel weighting. The present study evaluates this design within the LiteStar backbone; it does not establish superiority over all existing illumination-adaptive or attention modules.

LGD-Conv begins with a channel projection of input feature *X*:

(9) $$X^{\prime }=Conv_{1\times 1}\left(X\right).$$

The projected feature is processed along three paths:

(10) $$f_{loc}=DWConv_{3\times 3}\left(X^{\prime }\right),$$

(11) $$f_{sur}=Up\;\left(DWConv_{3\times 3}\left(AvgPool_{2\times 2}\left(X^{\prime }\right)\right)\right),$$

(12) $$f_{res}=Up\;\left(Conv_{1\times 1}\left(AvgPool_{2\times 2}\left(X^{\prime }\right)\right)\right).$$

The local path retains fine texture. Pooling enlarges the context of the surrounding path, while the residual path preserves low-frequency content. After upsampling, the outputs are concatenated:

(13) $$f_{joint}=PReLU\left(BN\left(Conv_{1\times 1}\left([f_{loc},f_{sur},f_{res}]\right)\right)\right).$$

Global illumination context then produces channel weights:

(14) $$\alpha =\sigma \left(FC_{2}\left(FC_{1}\left(GAP(f_{joint})\right)\right)\right),$$

where GAP(·) is global average pooling, FC(·) is a fully connected layer, and σ(·) is the sigmoid function. LGD-Conv returns to

(15) $$Y=\alpha ⊙f_{joint}+f_{res},$$

where ⊙ denotes channel-wise multiplication. The weighting changes with the frame-level illumination context, while the residual term limits information loss.

**Figure 4:**
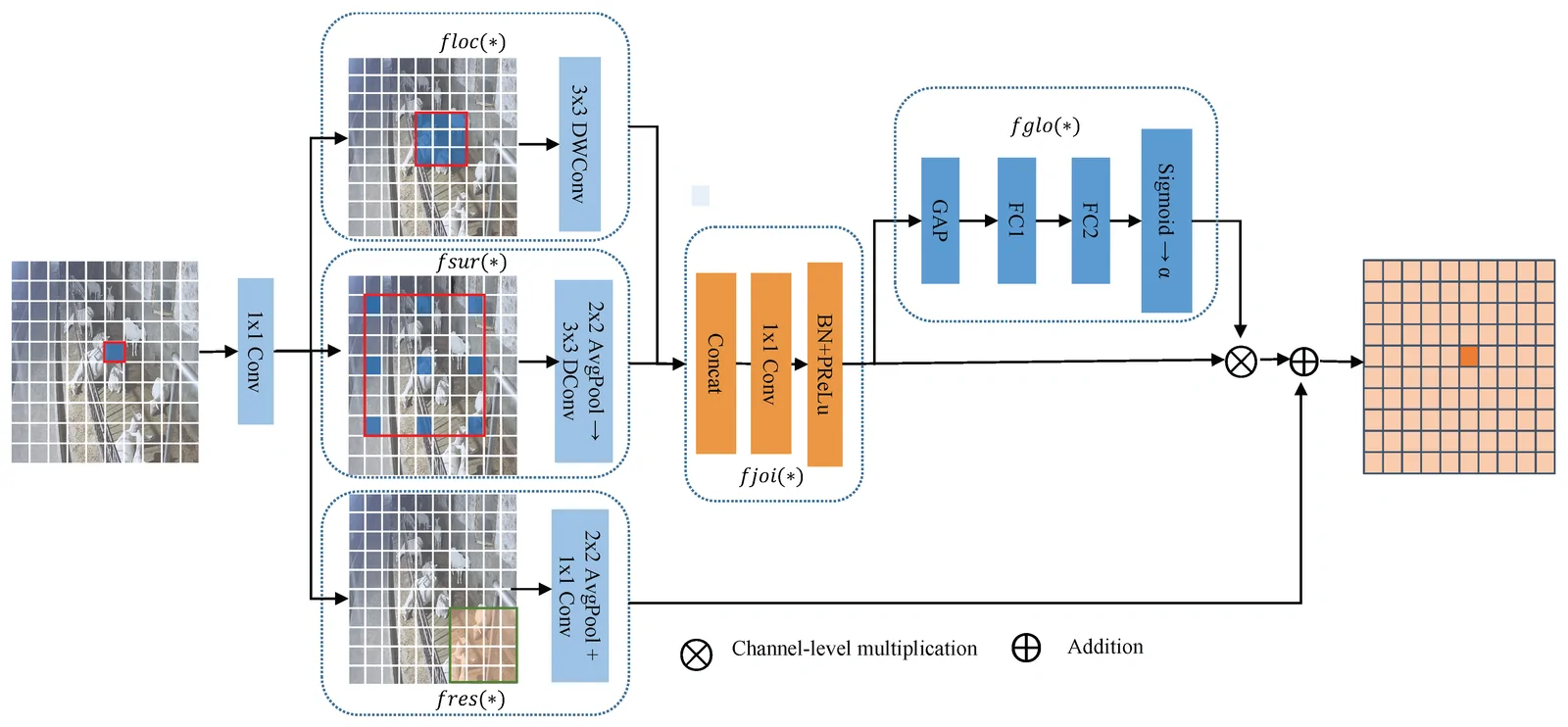
Structure of the LGD-Conv module. Local and pooled paths form a joint feature. Global illumination weights modulate this feature before residual addition. The asterisk (*) in labels such as $f_{\mathrm{loc}}\left(*\right)$, $f_{\mathrm{sur}}\left(*\right)$, $f_{\mathrm{res}}\left(*\right)$, $f_{\mathrm{joi}}\left(*\right)$, and $f_{\mathrm{glo}}\left(*\right)$ denotes the corresponding feature tensor at that processing stage and is used as a schematic placeholder rather than a multiplication operator.

The structure of the LGD-Conv module is illustrated in Figure 4.

### 3.5. Geometry-Aware Depth Branch

The depth branch processes the DA3 prediction with a compact YOLO11n-style backbone. Its role is geometric: it encodes relative distance, scene layout, and foreground separation rather than color or texture.

The depth branch outputs three feature maps:

(16) $$D_{l}=\Phi_{d}^{l}\left(I^{d}\right),\;l\in \left\{3,4,5\right\},$$

where $D_{3}$, $D_{4}$, and $D_{5}$ have strides of 8, 16, and 32. DRGF evaluates these features before they interact with the RGB stream.

### 3.6. Depth Reliability-Guided Fusion

**Figure 5:**
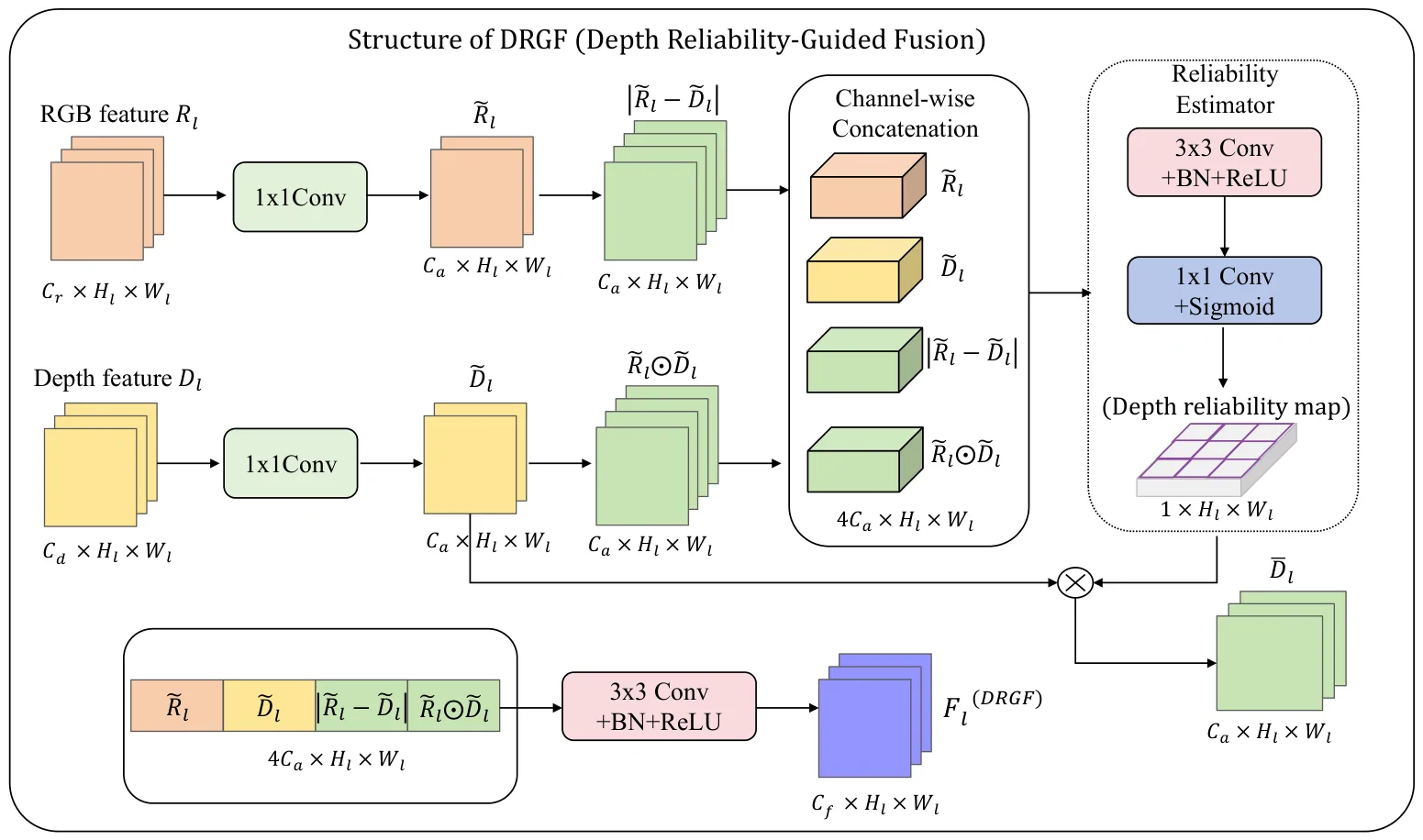
Structure of the depth reliability-guided fusion module. The estimator combines aligned RGB and depth features with their difference and product. Its output weights the depth feature before fusion.

DA3 predictions are informative but not uniformly reliable for the downstream detection task. Errors or ambiguous responses may occur near occlusions, saturated regions, reflective surfaces, fine railings, or areas with weak texture. DRGF therefore predicts a spatial fusion gate before the RGB and pseudo-depth features are combined. In this study, the term reliability map denotes an operational, task-learned weighting function rather than a calibrated estimate of physical depth accuracy.The gate was optimized only through the detection objective; it was not supervised with ground-truth depth, calibrated as a probability, or derived from the native DA3 confidence output. The architecture of the DRGF module is shown in Figure 5.

At pyramid level *l*, 1×1 convolutions align the channel dimensions of $R_{l}$ and $D_{l}$:

(17) $${\tilde{R}}_{l}=Conv_{1\times 1}\left(R_{l}\right),$$

(18) $${\tilde{D}}_{l}=Conv_{1\times 1}\left(D_{l}\right).$$

Difference and product features describe disagreement and local interaction:

(19) $$M_{l}^{diff}=\left|{\tilde{R}}_{l}-{\tilde{D}}_{l}\right|,$$

(20) $$M_{l}^{prod}={\tilde{R}}_{l}⊙{\tilde{D}}_{l}.$$

A convolution estimates reliability from the aligned and derived features:

(21) $$Q_{l}=\sigma \left(Conv\left([{\tilde{R}}_{l},{\tilde{D}}_{l},M_{l}^{diff},M_{l}^{prod}]\right)\right),$$

where $Q_{l}\in R^{1\times H_{l}\times W_{l}}$ is the learned spatial gating map. It weights the aligned depth feature:

(22) $${\bar{D}}_{l}=Q_{l}⊙{\tilde{D}}_{l}.$$

The preliminary fused feature is

(23) $$F_{l}^{DRGF}=Conv\left([{\tilde{R}}_{l},{\bar{D}}_{l},M_{l}^{diff},M_{l}^{prod}]\right).$$

Thus, a strong depth response contributes only when its learned gate is also high. Inconsistent cross-modal responses are reduced before later attention and pyramid aggregation. The detector ablation tests whether this gating operation improves detection, but it does not demonstrate that high $Q_{l}$ corresponds to physically more accurate depth.

### 3.7. Geometry-Guided Cross-Modal Attention

**Figure 6:**
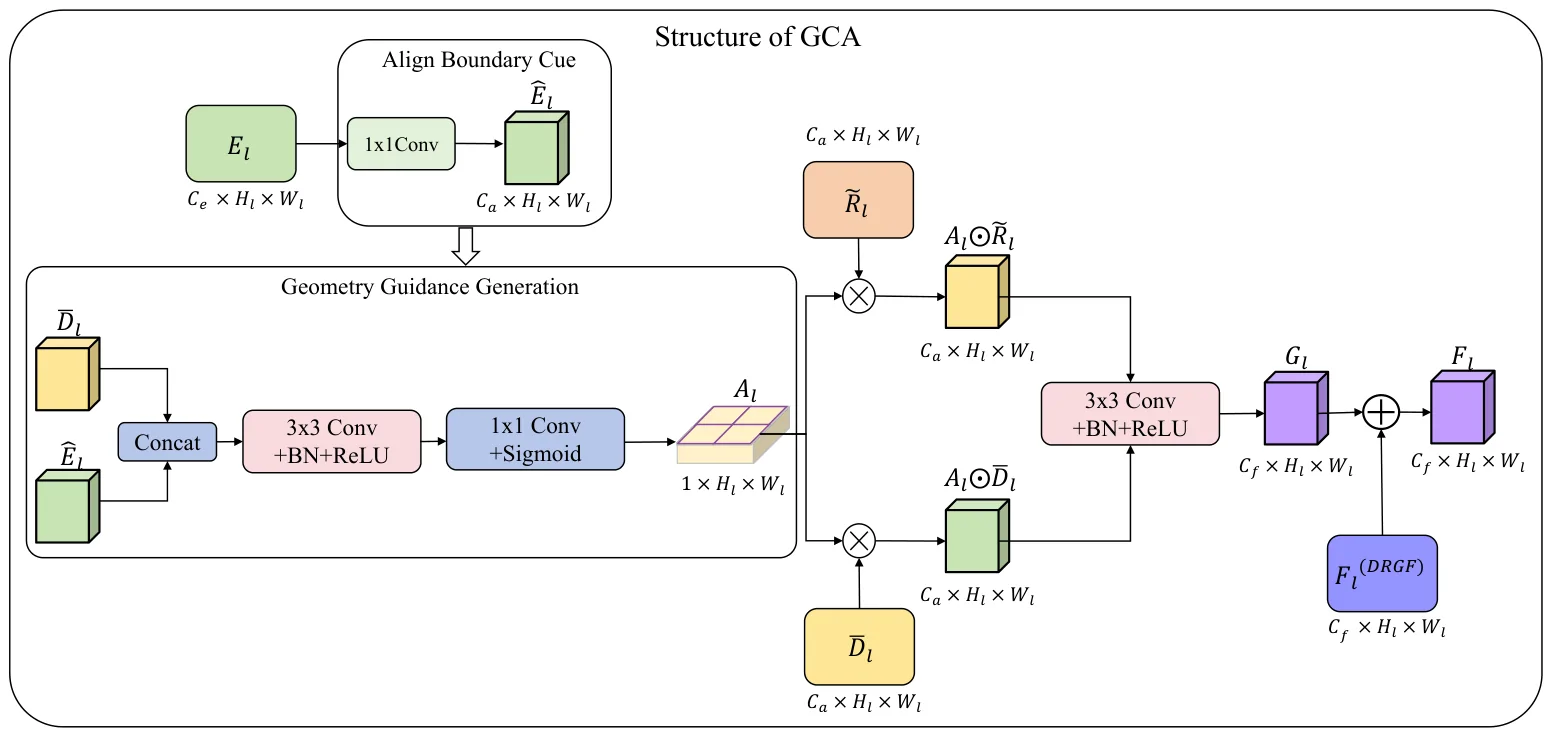
Simplified structure of the geometry-guided cross-modal attention module. A geometry attention map is estimated from the weighted depth feature and aligned boundary cue. It modulates both modalities before residual fusion.

Reliability weighting does not explicitly identify the contour between two adjacent goats. GCA adds this information after DRGF. It forms an attention map from the weighted depth feature and the corresponding boundary cue.The structure of the GCA module is presented in Figure 6.

A 1×1 convolution first aligns boundary cue $E_{l}$:

(24) $${\hat{E}}_{l}=Conv_{1\times 1}\left(E_{l}\right).$$

The geometry attention map is then

(25) $$A_{l}=\sigma \left(Conv\left([{\bar{D}}_{l},{\hat{E}}_{l}]\right)\right),$$

where $A_{l}\in R^{1\times H_{l}\times W_{l}}$. The same spatial map modulates both modalities:

(26) $$R_{l}^{att}=A_{l}⊙{\tilde{R}}_{l},$$

(27) $$D_{l}^{att}=A_{l}⊙{\bar{D}}_{l}.$$

The attended features are concatenated and fused:

(28) $$G_{l}=Conv\left([R_{l}^{att},D_{l}^{att}]\right).$$

A residual connection preserves the preliminary DRGF representation:

(29) $$F_{l}=G_{l}+F_{l}^{DRGF}.$$

The resulting $F_{l}$ becomes the input to DBE-BiFPN at level *l*. Adding GCA increased the cumulative parameter count from 3.70 M for the DRGF variant to 3.90 M (The cumulative changes are reported in Table 6). Per-variant latency was not recorded, so this parameter difference describes model-size overhead but does not isolate the timing cost of GCA.

### 3.8. Depth-Boundary-Enhanced BiFPN

DBE-BiFPN extends weighted bidirectional aggregation by adding a boundary input at every pyramid level. It receives fused features $\left\{F_{3},F_{4},F_{5}\right\}$ and the resized cues $\left\{E_{3},E_{4},E_{5}\right\}$. P3 retains fine contours for small or partly visible animals, whereas P4 and P5 combine coarser object-scale context for larger or strongly overlapping bodies. Supplying the boundary cue at all three levels is intended to prevent it from being available only before repeated downsampling and aggregation. A level-specific boundary ablation was not performed, so this is a design rationale rather than evidence that three-level injection is optimal.

**Figure 7:**
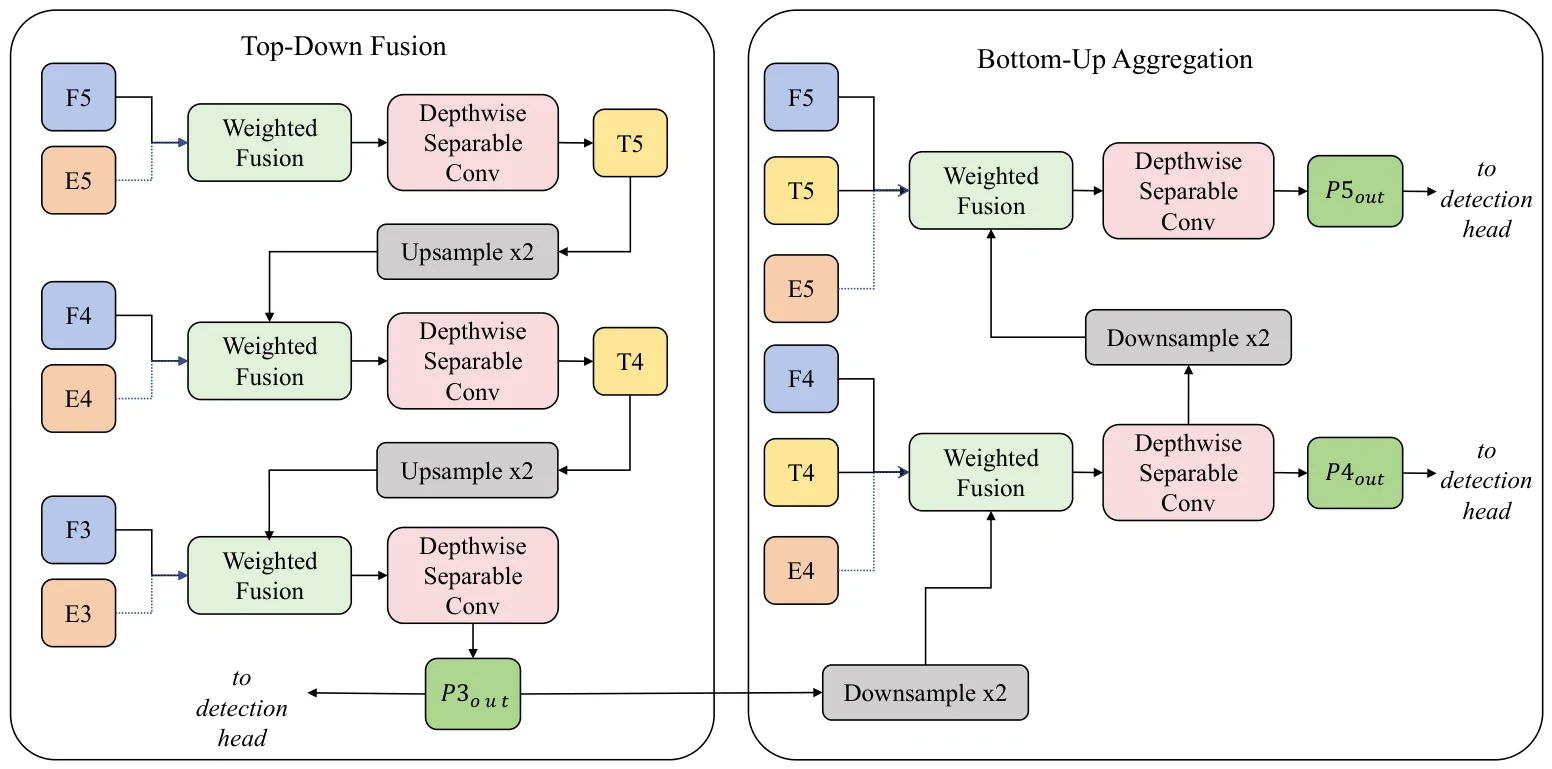
Simplified structure of the DBE-BiFPN neck. The top-down and bottom-up paths exchange semantic and spatial information. A projected depth boundary cue enters each pyramid level.

The structure of the DBE-BiFPN neck is shown in Figure 7.

For a weighted fusion node with *n* input features ${\left\{X_{i}\right\}}_{i=1}^{n}$, the output is calculated as

(30) $$W\left(X_{1},\ldots ,X_{n}\right)=\frac{\sum_{i=1}^{n}ReLU\left(w_{i}\right)X_{i}}{\sum_{i=1}^{n}ReLU\left(w_{i}\right)+\epsilon },$$

where $w_{i}$ is learnable and ϵ prevents division by zero. A depthwise separable convolution follows weighted fusion:

(31) $$B\left(X_{1},\ldots ,X_{n}\right)=DSConv\left(W(X_{1},\ldots ,X_{n})\right).$$

Here, DSConv(·) consists of depthwise and pointwise convolutions. Each boundary cue is projected to the feature dimension before summation:

(32) $${\tilde{E}}_{l}=Conv_{1\times 1}\left(E_{l}\right),\;l\in \left\{3,4,5\right\}.$$

The top-down path transfers coarse semantic information toward P3:

(33) $$T_{5}=B\left(F_{5},{\tilde{E}}_{5}\right),$$

(34) $$T_{4}=B\left(F_{4},Up\left(T_{5}\right),{\tilde{E}}_{4}\right),$$

(35) $$P_{3}^{out}=B\left(F_{3},Up\left(T_{4}\right),{\tilde{E}}_{3}\right),$$

where Up(·) denotes two-fold upsampling. The bottom-up path returns finer spatial information to P4 and P5:

(36) $$P_{4}^{out}=B\left(F_{4},T_{4},Down\left(P_{3}^{out}\right),{\tilde{E}}_{4}\right),$$

(37) $$P_{5}^{out}=B\left(F_{5},T_{5},Down\left(P_{4}^{out}\right),{\tilde{E}}_{5}\right),$$

where Down(·) denotes two-fold downsampling. The detection head receives $\left\{P_{3}^{out},P_{4}^{out},P_{5}^{out}\right\}$.

### 3.9. Implementation Details and Reproducibility

All detector inputs were resized to 640 × 640 pixels. For each RGB image, the frozen DA3-Small model generated one single-channel relative pseudo-depth map offline. Each pseudo-depth map was independently normalized to [0,1] using min–max scaling with $\epsilon =10^{-6}$. Because the pseudo-depth map was generated directly from the corresponding RGB frame, no additional geometric registration between the two detector inputs was required.

Both feature branches produced P3, P4, and P5 representations with strides of 8, 16, and 32, respectively. The boundary pathway applied horizontal and vertical Sobel filtering to the normalized pseudo-depth map and calculated the gradient magnitude before resizing it to the three pyramid resolutions. In DRGF and GCA, 1×1 convolutions were used for cross-modal channel alignment and boundary projection. Both $Q_{l}$ in DRGF and $A_{l}$ in GCA were single-channel sigmoid spatial maps with dimensions $1\times H_{l}\times W_{l}$. DBE-BiFPN injected the resized boundary representation at P3, P4, and P5 and applied normalized learnable fusion weights followed by depthwise separable convolution.

The equations in the method subsections use the generic notation Conv(·) to emphasize information flow rather than to reproduce the complete layer configuration. The exact layer-wise channel widths, block repeat counts, kernel sizes, strides, activation functions, and module arguments used for the reported experiments are specified in the released model configuration files and source code. These files should be used together with the training settings in Table 2 to reproduce the reported architecture and computational complexity. The primary comparison used random seed 42. To quantify training variability in the cumulative ablation, all six configurations were additionally retrained with seeds 0, 21, 42, 3407, and 2026 under the same split and hyperparameters. The robustness summaries report the arithmetic mean and sample standard deviation (SD, denominator n−1) across the five runs; the seed-42 values remain the point estimates used in the main detector comparison. Detector-speed measurements used FP16 inference, batch size 1, 100 warm-up iterations, and 1000 timed runs on an NVIDIA RTX 4090. For dual-stream models, these timing measurements began after the corresponding pseudo-depth maps had been generated.

## 4. Experiments and Results

### 4.1. Dataset Preparation and Experimental Setup

Experiments used images collected at the NWAFU teaching farm. The source corpus contains variation in illumination, animal density, viewpoint, and occlusion, and railings frequently cross goat bodies. Each visible animal was manually labeled with a rectangular box under the single class *goat*. The source corpus comprised 1521 images and 13,985 bounding boxes. Because all images came from one farm and one Saanen herd, this variation should not be interpreted as biological or cross-site diversity.

A fixed seed of 42 was used to partition the 1521 source images into 1065 training, 304 validation, and 152 test images. Source-video identifiers were used to ensure that no video represented in training also contributed images to the test subset. Augmented variants were generated only after partitioning and remained with their corresponding training source. The final source images were obtained from 248 videos recorded on 15 collection days between 15 March and 1 April 2026 after video-wise pHash de-duplication ($d_{H}\leq 6$), group-wise sharpness selection (maximum Laplacian variance with a minimum accepted value of 100), and manual quality control; therefore, they did not form contiguous frame sequences. The test subset was held out from model fitting, and the reported test metrics are not affected by direct source-video overlap with the training subset. The available records do not establish whether the validation subset was source-video-disjoint from both training and test. The split was also not grouped by individual animal identity or collection day; repeated appearances of the same goats and day-specific conditions may therefore remain across validation and another subset. This limitation affects the strength of generalization claims and is stated explicitly below.

Only training images were augmented. Each was retained in its original form and supplemented with Gaussian blur, Gaussian noise, grayscale conversion, salt-and-pepper noise, histogram equalization, random saturation adjustment, and random contrast adjustment. These photometric operations preserved bounding-box geometry. The resulting training set contained 8520 image files and 77,600 annotations. Validation and test images remained unaugmented. Thus, the 12,168-image public archive describes all source-level variants, whereas the experiments used 8976 images: 8520 augmented-or-original training images, 304 original validation images, and 152 original test images.

The frozen DA3-Small checkpoint produced a paired pseudo-depth map for every RGB image supplied to the detector. Both inputs were resized to the same resolution. Each depth map was independently normalized to [0,1] by min–max scaling with $\epsilon =10^{-6}$. The test set was held out until final evaluation. Pretrained weights and label-preserving augmentation increase training exposure, while the common split enables controlled comparison among detector variants. They do not compensate for the modest number of independent source images or the absence of external validation. Accordingly, the experiment is interpreted as a within-farm architecture comparison rather than as a population-level validation study. Table 3 distinguishes the source-level partition from the images used by the detector.

All models followed the same training and evaluation protocol. Inputs were 640 × 640 pixels, and official pretrained weights were used when available. Table 4 lists the remaining settings. The primary run used seed 42. For the six cumulative ablation configurations, we repeated training with seeds 0, 21, 42, 3407, and 2026 while keeping the data split, augmentation, initialization, optimizer, and stopping schedule unchanged. We report mean ± sample SD for these repeated runs and retain the seed-42 values in the main comparison tables for consistency with the originally reported benchmark. The ablation repeat therefore quantifies training variability for the proposed component sequence, whereas the comparisons with mainstream detectors and representative fusion strategies remain single-run point estimates. Speed measurements used FP16, batch size 1, 100 warm-up iterations, and 1000 timed runs. Latency for dual-stream detectors excludes offline DA3 generation.

**Figure 8:**
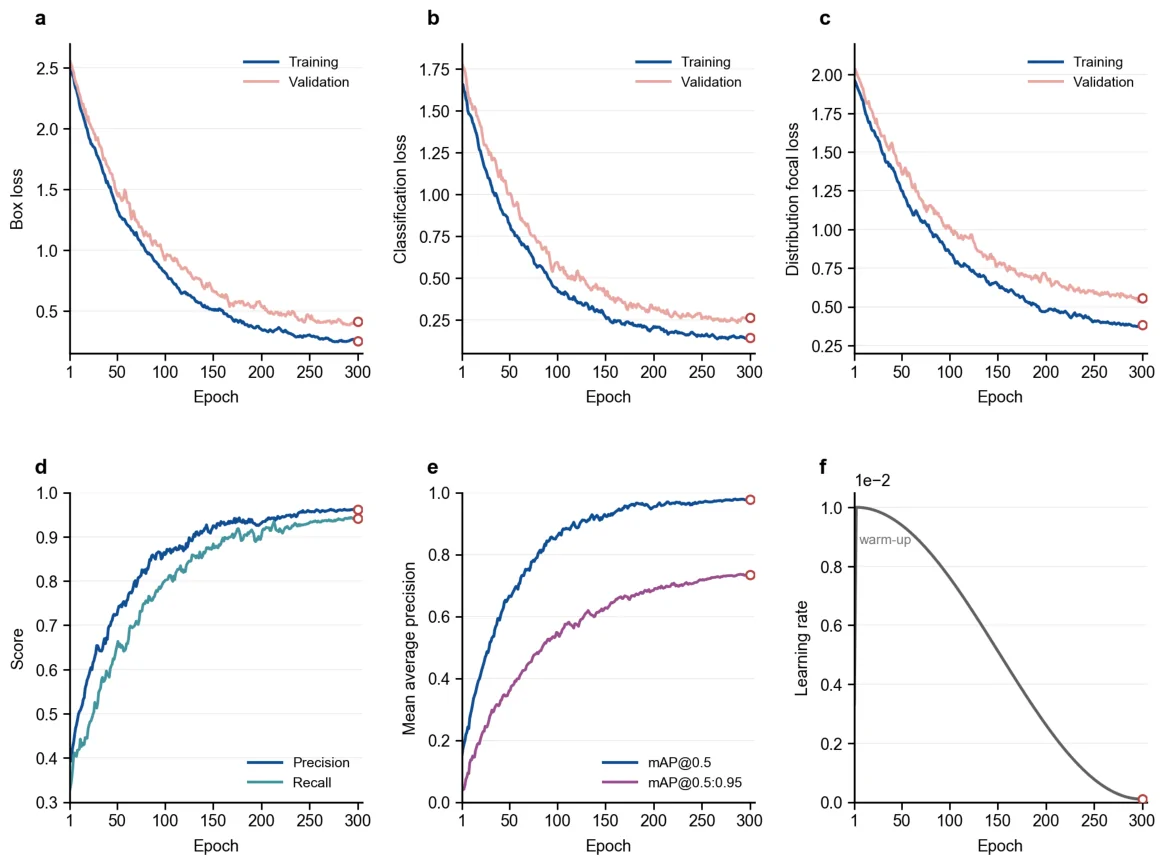
Training dynamics of DRG-LiteStar-YOLO over 300 epochs: (**a**) box loss; (**b**) classification loss; (**c**) distribution focal loss; (**d**) precision and recall; (**e**) mAP@0.5 and mAP@0.5:0.95; (**f**) learning-rate schedule. Blue and pink curves in panels (**a**–**c**) show training and validation losses. Red-outlined markers indicate the values recorded at epoch 300.

The optimization history is shown in Figure 8. Box, classification, and distribution focal losses fell sharply during the early stage, followed by a slower decline as the learning rate decayed. The validation curves remained above their training counterparts. They nevertheless continued to improve late in training, reaching their minima at epochs 290, 287, and 296, respectively. Small rebounds occurred in the last few epochs, but they were local fluctuations rather than sustained divergence.

Precision remained at or above 95% of its terminal value from epoch 148 onward, while mAP@0.5 reached this stable region at epoch 158. Recall stabilized later, at epoch 200. The stricter mAP@0.5:0.95 measure required 227 epochs, indicating that coarse object recognition converged before high-IoU localization. Over the final 20 epochs, the mean mAP@0.5:0.95 was 0.7335 with a standard deviation of 0.0019. This describes within-run variation across successive checkpoints, not variation across independent training seeds, and should not be used as a confidence interval for model performance. The trajectory had entered a narrow plateau before the final checkpoint.

In the primary seed-42 run, precision at epoch 300 was 0.961, recall was 0.941, mAP@0.5 was 0.978, and mAP@0.5:0.95 was 0.735. The corresponding training losses were 0.249, 0.143, and 0.382 for box regression, classification, and distribution focal loss. Validation losses were 0.410, 0.263, and 0.556. These endpoint values are marked in Figure 8; all curves are plotted directly from the recorded 300-epoch log.

### 4.2. Evaluation Metrics

Detection quality was measured by precision, recall, and mean average precision. We also report parameter count, floating-point operations, latency, and throughput. Precision and recall are defined as

(38) $$Precision=\frac{TP}{TP+FP},$$

(39) $$Recall=\frac{TP}{TP+FN},$$

where TP, FP, and FN denote true positives, false positives, and false negatives, respectively.

Average precision is obtained from the precision–recall curve. We report mAP@0.5 and the stricter mAP@0.5:0.95, averaged over IoU thresholds from 0.5 to 0.95 in steps of 0.05. Parameter count and FLOPs describe model complexity. Frames per second (FPS) and latency describe detector speed.

### 4.3. Comparison with Mainstream Detection Models

We compared DRG-LiteStar-YOLO with Faster R-CNN, SSD, and several compact YOLO models. Every detector used the same source-level split, augmented training data, and input resolution. Table 5 reports the results on the held-out, unaugmented test set.

DRG-LiteStar-YOLO had the highest point-estimate mAP values in Table 5. Relative to YOLO11n, mAP@0.5 increased from 0.954 to 0.978. The corresponding change in mAP@0.5:0.95 was larger, from 0.675 to 0.735. The detector contained 4.20 M parameters and required 12.60 GFLOPs. Throughput was 86.2 FPS when depth maps were precomputed; DA3 preprocessing was not included in this measurement. These mainstream-detector comparisons used the primary seed-42 run and were not subjected to the five-seed repeat; the replicated cumulative ablation is reported separately below.

### 4.4. Ablation Study

The ablation sequence began with YOLO11n. We then replaced its RGB backbone with LiteStar-YOLO and LGD-Conv. The depth branch, DRGF, GCA, and DBE-BiFPN were added in that order. Table 6 records the cumulative changes.

Replacing the baseline backbone with LiteStar-YOLO and LGD-Conv reduced the parameter count from 2.60 M to 2.10 M, while mean mAP@0.5:0.95 increased from 0.676±0.004 to 0.697±0.004. The depth branch raised the mean to 0.709±0.004, and DRGF produced the largest later increment, reaching 0.724±0.004. GCA increased the mean to 0.732±0.003, whereas DBE-BiFPN produced a smaller final increase to 0.734±0.002. Recall increased at every step, from 0.910±0.003 in YOLO11n to 0.941±0.003 in the full model. The mean trend was consistent across the five seeds, and the small standard deviations indicate limited training-run variability for this cumulative sequence. Because latency was recorded only for selected complete configurations, the timing cost of each module cannot be separated.

### 4.5. Cross-Seed Robustness of the Cumulative Ablation

**Figure 9:**
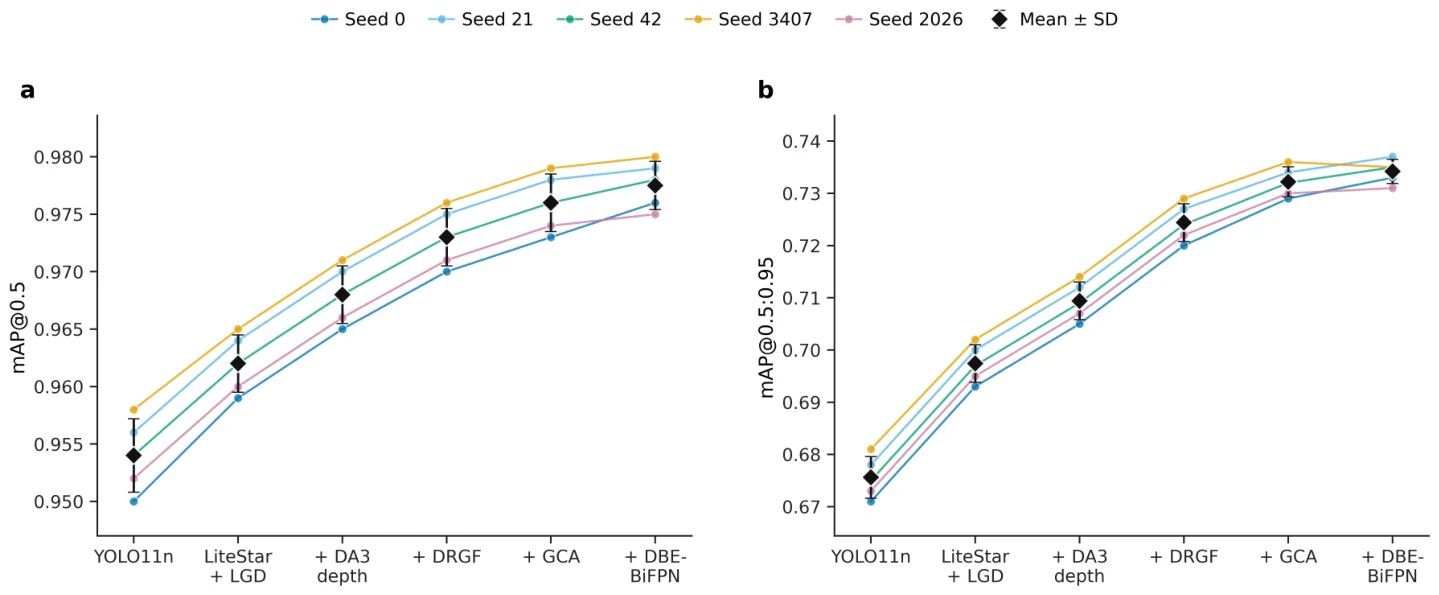
Five-seed variability of the cumulative ablation configurations. Individual colored points show independent training runs with seeds 0, 21, 42, 3407, and 2026; lines connect results from the same seed. Black diamonds and error bars denote the mean and sample SD across five runs. (**a**) mAP@0.5. (**b**) mAP@0.5:0.95.

Table 7 and Figure 9 summarize the five-seed check using the same held-out test partition. Individual points show the five independent runs, and the black diamonds show the mean ± SD. The RGB-only LiteStar configuration achieved 0.697±0.004 mAP@0.5:0.95, whereas the full model achieved 0.734±0.002. The paired full-model gain over LiteStar was positive for all five seeds and averaged 0.0368, indicating that the principal pseudo-depth advantage was not dependent on seed 42. The final DBE-BiFPN increment was smaller, 0.0020 on average, and was positive in four of the five raw runs; it should therefore be interpreted as a modest and more weakly separated contribution rather than as the main source of the overall gain. These results support the stability of the cumulative architecture trend while leaving the single-run status of the external detector and fusion-strategy comparisons unchanged.

### 4.6. Effect of Pseudo-Depth and Fusion Configuration

Table 8 compares single-modality inputs, conventional fusion strategies, and the complete proposed configuration. Early input fusion, direct feature concatenation, and generic attention were selected as representative low-, intermediate-, and adaptive-fusion baselines. They do not constitute an exhaustive survey of multimodal architectures. The last row includes DRGF, GCA, and DBE-BiFPN. All configurations used the same LiteStar backbone and training protocol.

### 4.7. Integrated Performance Visualization

**Figure 10:**
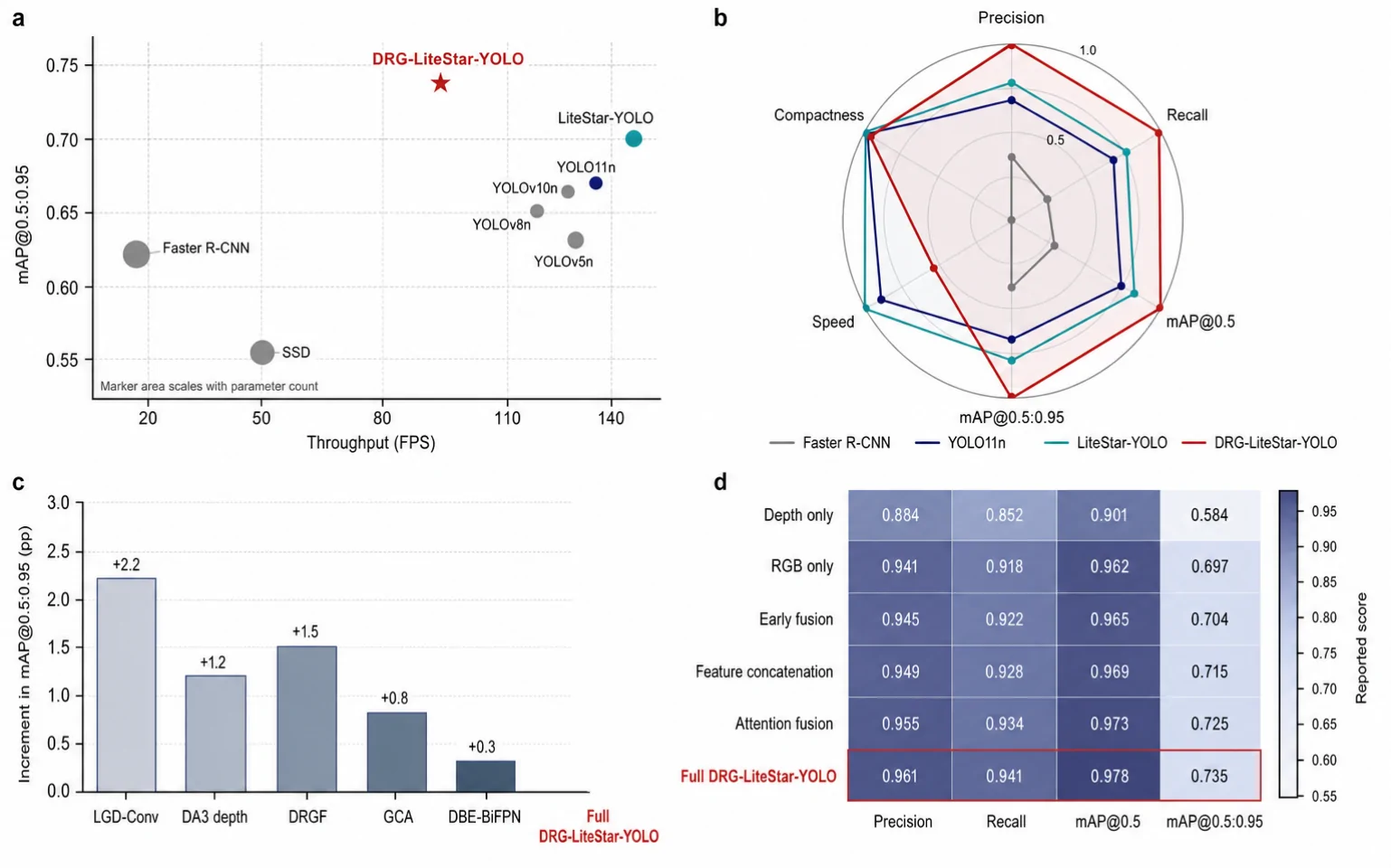
Integrated visualization of detection performance and component contributions. (**a**) Accuracy–throughput comparison across eight detectors; marker area represents parameter count. (**b**) Radar profiles of four representative models. Precision, recall, mAP@0.5, mAP@0.5:0.95, and speed were min–max normalized across all eight detectors, while compactness was inversely normalized from parameter count. (**c**) Incremental improvement in mAP@0.5:0.95 after each model component was introduced. (**d**) Performance heatmap for the evaluated modalities and representative multimodal configurations. Values are the point estimates reported in Table 5, Table 6, Table 7 and Table 8; cross-seed variability was evaluated separately for the cumulative ablation in Table 6.

Figure 10 combines the numerical comparisons from Table 5, Table 6, Table 7 and Table 8. In the accuracy–throughput panel, DRG-LiteStar-YOLO reached 0.735 mAP@0.5:0.95 at 86.2 FPS. The compact RGB detectors were faster. LiteStar-YOLO, for example, reached 145.7 FPS with 2.10 M parameters, but its mAP@0.5:0.95 was 0.697. The radar plot displays this difference directly: DRG-LiteStar-YOLO scored higher on the four detection metrics, while LiteStar-YOLO and YOLO11n were faster and smaller.

Panel (c) reports the cumulative change after each component was introduced. LGD-Conv added 2.2 percentage points to mAP@0.5:0.95, followed by 1.2 points from the depth branch. Among the later additions, DRGF contributed 1.5 points, GCA 0.8 points, and DBE-BiFPN 0.3 points. The configuration heatmap compares the tested inputs, conventional fusion schemes, and the complete proposed model. Depth-only input gave the lowest mAP@0.5:0.95 in that experiment (0.584), well below the RGB-only value of 0.697.

### 4.8. Visualization Results

The visual comparison used three barn frames: one in daylight, one under low light, and one at night. Faster R-CNN, YOLOv8n, YOLO11n, LiteStar-YOLO, and DRG-LiteStar-YOLO processed the same images. Feature-response maps were examined separately from the bounding-box outputs.

#### 4.8.1. Qualitative Comparison of Detection Results

**Figure 11:**
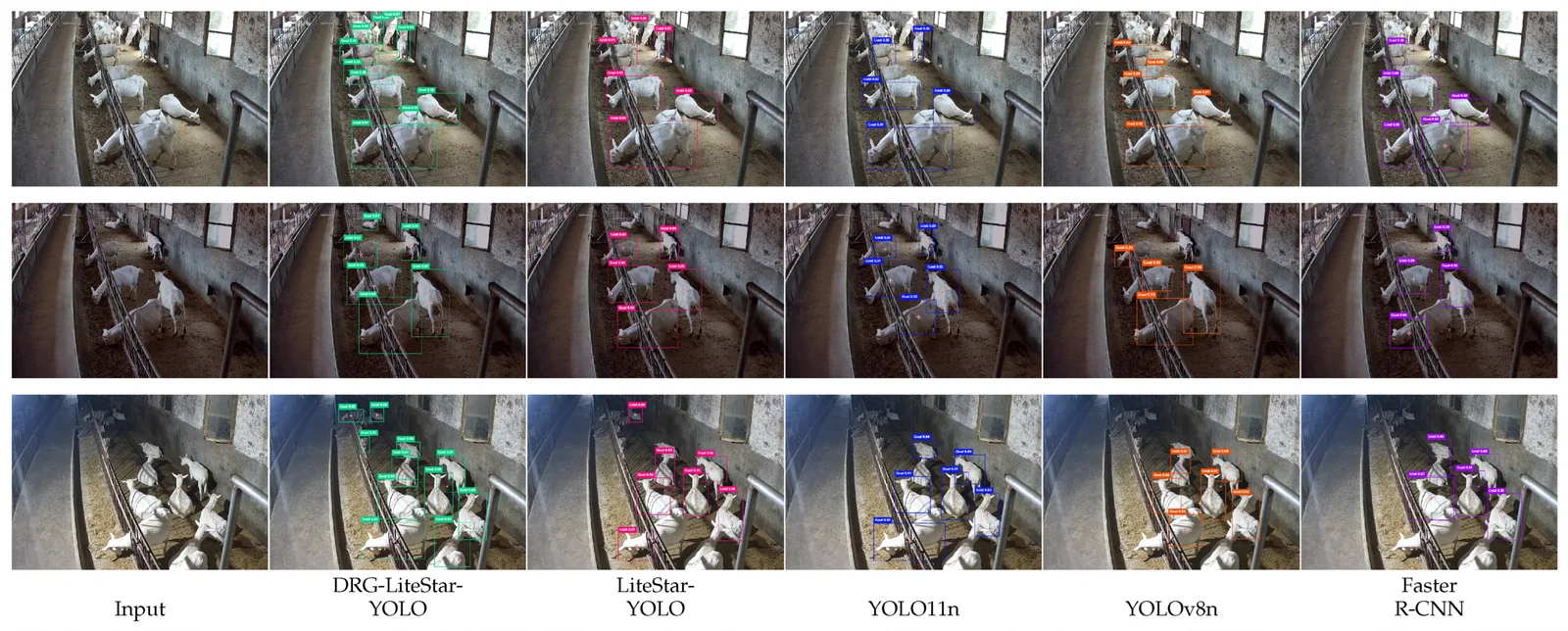
Qualitative comparison of detection results under different illumination conditions. Rows show daylight, low-light, and night scenes. Columns show the input and detections from DRG-LiteStar-YOLO, LiteStar-YOLO, YOLO11n, YOLOv8n, and Faster R-CNN.

Figure 11 presents the three comparisons. Columns show the input followed by DRG-LiteStar-YOLO, LiteStar-YOLO, YOLO11n, YOLOv8n, and Faster R-CNN. Rows correspond to daylight, low light, and night scenes.

All five detectors found most goats in the daylight frame. Their outputs differed mainly at the railing, near the image boundary, and where two bodies overlapped. In this example, DRG-LiteStar-YOLO retained boxes around more of the partly interrupted animals.

The low-light frame contained weaker contrast between white coats and the surrounding floor and walls. Several comparison models missed partly visible goats or covered only part of the body. DRG-LiteStar-YOLO returned more detections in the same regions.

The night frame combined saturated areas around the lamps with deep shadows. Missed detections were concentrated on animals that crossed these two lighting zones. DRG-LiteStar-YOLO detected more of the visible goats in this frame. These three examples show where the models differed, but they do not measure how often each failure occurred across the test set.

#### 4.8.2. Failure Modes and Scope of the Qualitative Evidence

The qualitative examples in Figure 11 were selected to illustrate challenging daylight, low-light, and night conditions and are predominantly successful detection examples. They should therefore not be interpreted as a systematic failure-case benchmark. During dataset construction, frames with substantial truncation, inseparable severe overlap, global blur, or extreme illumination were removed when individual goats could no longer be annotated consistently. As a result, the most severe forms of these conditions are under-represented in the evaluation set. This sampling choice improves annotation consistency but also means that the current evaluation cannot provide a representative frequency estimate for extreme failure modes under deployment conditions.

Potential failure conditions nevertheless remain. Body truncation, motion blur, reflective surfaces, lamp saturation, severe occlusion, and weak separation between adjacent animals may degrade both RGB appearance and pseudo-depth boundaries. The current test set was not stratified according to these factors, and false negatives, false positives, and poor-localization cases were not quantified separately for each condition. Accordingly, the qualitative examples are used only to illustrate representative model behavior and not to estimate the frequency of particular failure modes. A future error-stratified evaluation based on prespecified detection-error criteria is required to quantify robustness under these conditions.

#### 4.8.3. Feature Response Heatmap Analysis

**Figure 12:**
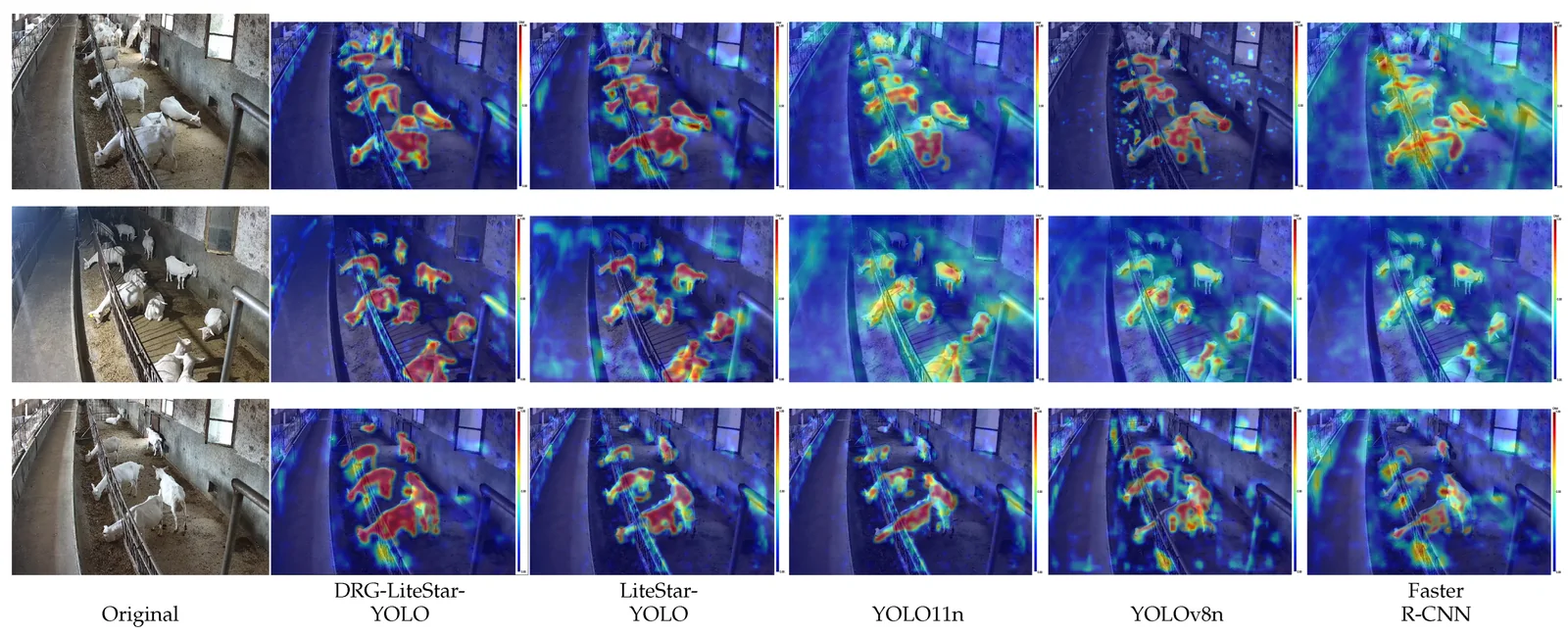
Comparison of feature-response heatmaps generated by different detection models on three randomly selected test images. Rows correspond to the three samples. Columns show the input and heatmaps from DRG-LiteStar-YOLO, LiteStar-YOLO, YOLO11n, YOLOv8n, and Faster R-CNN. Warmer regions indicate stronger model responses.

Three test images were randomly selected for feature-response visualization. Every model used the same images. Grad-CAM was applied to the final convolutional stage feeding each detection head, with the target defined as the summed confidence of retained goat detections. Heatmaps were independently normalized to [0,1] and rendered with identical color settings. Figure 12 shows the resulting grid.

Faster R-CNN, YOLOv8n, and YOLO11n responded to most visible goat regions. Their high-response areas also extended onto railings and other strong edges, particularly where a body contour was interrupted.

LiteStar-YOLO concentrated more of its response on the animals, although several overlapping or weak-texture regions remained fragmented. DRG-LiteStar-YOLO produced broader connected responses over the same bodies. In the selected frames, less activation extended onto the surrounding railings.

Because each heatmap was normalized independently, color intensity should be interpreted within an image rather than as an absolute response magnitude across models. The three selected samples provide descriptive evidence of differences in spatial feature response, but they do not establish that one model has quantitatively better feature localization over the complete test set. In addition, the visualization does not isolate the contribution of any individual proposed module. No pointing-game score, activation–annotation overlap metric, or attribution-stability analysis was calculated. The Grad-CAM results are therefore treated as qualitative diagnostic evidence rather than as quantitative or causal validation of the proposed fusion mechanism.

### 4.9. Model Complexity and Inference Efficiency

Table 9 compares detector size and speed. The measurements include parameters, FLOPs, model size, latency, and FPS.

The second stream and fusion modules increased detector cost relative to LiteStar-YOLO. Depthwise separable convolutions kept the detector at 4.20 M parameters, and throughput reached 86.2 FPS after depth maps were available. This is detector-only speed, not end-to-end throughput. An online system would also incur DA3 inference, image transfer, normalization, and scheduling time; because those terms were not measured, real-time operation of the complete pipeline is not demonstrated.

## 5. Discussion

### 5.1. Interpreting Reliability-Guided Pseudo-Depth Fusion

The main within-run finding is not simply that adding a second image representation improved detection. Pseudo-depth alone was weaker than RGB, reaching 0.584 mAP@0.5:0.95 compared with 0.697±0.004 for the RGB-only LiteStar model in the five-seed ablation. Its benefit appeared when RGB remained the primary appearance signal, and depth was treated as auxiliary geometric evidence. The repeated cumulative sequence preserved the same ordering: the RGB-only LiteStar, depth-branch, DRGF, GCA, and full configurations reached mean mAP@0.5:0.95 values of 0.697, 0.709, 0.724, 0.732, and 0.734, respectively. Early fusion, direct feature concatenation, and generic attention produced single-run values of 0.704, 0.715, and 0.725, whereas the complete proposed configuration reached 0.735 in the seed-42 comparison. The five-seed results therefore strengthen the stability claim for the cumulative architecture, while not converting the alternative fusion comparison into a replicated experiment.

This ordering is consistent with a hypothesis based on uneven monocular-depth quality in barn images. Railings interrupt body contours, white coats often have little contrast against bright floors, and strong lamps create abrupt transitions between glare and shadow. A pseudo-depth map may retain useful relative structure in these regions, but it may also introduce false edges or distorted surfaces. Depth Anything models transfer relative geometric information rather than calibrated physical distance [24,25,26]. The fusion results suggest that learned attenuation of inconsistent depth is useful for detection, but they do not show that the DRGF gate is a calibrated indicator of depth accuracy.

DRGF and GCA address two different parts of this problem. DRGF reduces the influence of depth responses that disagree with RGB features. GCA uses the gated geometric signal to emphasize boundaries during cross-modal interaction. This division is related to depth calibration and cooperative fusion in RGB-D saliency detection [27,28,29,30], although goat detection requires bounding-box localization rather than pixel-level saliency. The cumulative ablation provides indirect task-level support for selective fusion. Independent comparison of $Q_{l}$ with true depth error, synthetic corruption, or calibrated confidence remains necessary to validate the reliability interpretation.

### 5.2. Boundary Cues and Localization in Crowded Barn Scenes

The larger point-estimate improvement occurred in the stricter localization metric. Relative to RGB-only LiteStar-YOLO, the full model increased mAP@0.5 from 0.962 to 0.978, a gain of 1.6 percentage points. The increase in mAP@0.5:0.95 was 3.8 points, from 0.697 to 0.735. Because the latter metric averages performance over progressively stricter IoU thresholds, this pattern is consistent with improved box placement rather than only easier object recognition. Per-IoU and cross-seed analyses would be needed to attribute the difference more precisely.

The component sequence provides a more detailed view. Across five seeds, adding the depth branch raised mean mAP@0.5:0.95 from 0.697 to 0.709, and DRGF produced the largest subsequent increment, to 0.724. GCA added 0.8 percentage points, while DBE-BiFPN added a further 0.2 points on average. GCA acts during fusion, whereas DBE-BiFPN carries depth-edge information through P3, P4, and P5. This differs from conventional feature-pyramid aggregation, which combines scale-dependent features without an explicit geometric boundary signal [18,19], and adapts multi-scale contour processing [31,32] to crowded bounding-box detection. The repeated results support a positive mean direction for the DBE-BiFPN increment, which was observed in four of five runs, but its small magnitude and the absence of a prespecified inferential test still warrant cautious interpretation.

The visual comparisons illustrate differences near railings, image borders, overlapping bodies, and abrupt lighting changes. In the three selected frames, DRG-LiteStar-YOLO retained more visible goats and produced more connected feature responses over several animals. These examples were selected for illustration, do not replace test-set error stratification, and do not show how frequently each outcome occurs. Independently normalized heatmaps also cannot be compared as absolute activation scores. They are qualitative diagnostics rather than quantitative evidence for a causal mechanism.

The practical case for pseudo-depth is therefore conditional rather than universal. In well-lit frames with separated animals and clear coat–floor contrast, the RGB-only LiteStar-YOLO baseline already provides strong detection and is the preferable configuration when minimum latency, memory, or edge-device cost is the primary requirement. The proposed two-stream model becomes more defensible when box placement must remain stable at stricter IoU thresholds and appearance boundaries are weakened by railings, partial overlap, image-border truncation, low contrast, or transitions between glare and shadow. These are the locations where the qualitative comparisons show the clearest differences from the RGB baseline, and they are consistent with the larger gain in mAP@0.5:0.95 than in mAP@0.5. Such localization can matter upstream of tracking or counting because loose, merged, or partial boxes can alter association and region-based measurements. However, downstream benefits were not evaluated, and the present test set was not stratified to estimate a separate gain for each condition. Accordingly, the pseudo-depth configuration should be preferred for server-side or cached-depth workflows in which boundary-sensitive localization justifies the additional computation; the RGB-only model remains the more appropriate default for uncomplicated scenes or tightly constrained online hardware.

### 5.3. Accuracy, Efficiency, and Use in Farm Monitoring

Comparison with prior livestock studies requires care because published systems use different species, labels, image sources, and test protocols. Earlier dairy goat work established surveillance-video detection with Faster R-CNN [7]; more recent studies address goat behavior recognition, goat tracking, and dairy cow detection–tracking rather than the same single-frame benchmark [5,6,12]. The present gain should therefore be interpreted against models retrained on the common NWAFU split, not as numerical superiority over results reported on other livestock datasets.

Within that protocol, the proposed model makes an accuracy–efficiency trade-off. Its point estimate for mAP@0.5:0.95 was 6.0 percentage points above YOLO11n and 3.8 points above LiteStar-YOLO, but it was slower than both RGB-only detectors. The two-stream architecture used 4.20 M parameters and 12.60 GFLOPs. With pseudo-depth maps prepared in advance, it processed 86.2 FPS on the RTX 4090, compared with 145.7 FPS for LiteStar-YOLO. The additional detector computation is associated with more accurate localization in this run rather than greater throughput.

Compatibility with ordinary RGB cameras is a practical advantage because the geometric signal is introduced through computation rather than a separate depth sensor. This shifts the deployment burden from camera hardware to depth estimation. The measured detector speed is relevant to offline analysis or pipelines that cache pseudo-depth, but it does not demonstrate real-time online monitoring. A live implementation must also include DA3 inference, image transfer, normalization, and scheduling overhead. The reported 86.2 FPS is therefore the capacity of the detector after depth generation, not the frame rate of the complete camera-to-output system or an edge device.

Detection is only one stage of animal monitoring. The present model locates goats in individual frames; it does not maintain identities, quantify behavior, infer emotion, assess health, or count animals across time. Its relevance to animal monitoring is therefore upstream and methodological: frame-level boxes can serve as observations for later association, trajectory analysis, counting, or behavior recognition, as in detection–tracking and behavior-analysis pipelines [5,6,12]. More accurate localization may provide a stronger input to those downstream tasks, but that implication was not tested here. Linking the detector to tracking and behavior models and evaluating task-level outcomes are necessary before claiming a behavioral, health, or welfare benefit.

### 5.4. Limitations and Future Work

The dataset defines the main boundary of the study. All 1521 source images came from 134 Saanen goats at one farm. Augmentation expanded photometric conditions and produced a 12,168-image release archive, but it did not add new animals, barn layouts, camera systems, or breeds. The de-duplication and quality-control process improved content diversity and annotation feasibility, but excluding frames with unidentifiable targets also narrowed evaluation under the most severe truncation, overlap, blur, and exposure conditions. Source-video identifiers prevented any of the 248 recordings from contributing to both training and test, which protects the final test metrics from direct train–test source-video overlap. Video-level disjointness involving validation was not verified, and the split was not grouped by individual animal or collection day, so the same recordings, goats, or day-specific environmental conditions may appear across validation and another subset. Cross-farm, cross-breed, cross-camera, animal-independent, day-independent, and fully video-disjoint evaluation is therefore necessary to determine how much of the reported gain transfers to other production settings. Transfer to other livestock species also cannot be assumed: differences in body morphology, coat texture, group density, housing structures, and camera geometry may change the usefulness of pseudo-depth and boundary cues and require species-specific validation.

Experimental uncertainty should also be examined more fully. The six cumulative ablation configurations were repeated across five prespecified seeds, and their mean ± SD values are now reported in Table 6 and Figure 9. This reduces, but does not eliminate, uncertainty because five seeds provide a descriptive estimate rather than a definitive population-level confidence interval, and no formal paired hypothesis test was prespecified. The comparisons with mainstream detectors and representative fusion strategies remain single-run point estimates, as do the latency measurements. The small DBE-BiFPN increment should therefore not be treated as independently established by the repeated ablation alone. A larger test set stratified by illumination, occlusion level, animal density, motion blur, reflection, and camera viewpoint would also convert the current illustrative examples into a quantitative failure analysis.

Finally, pseudo-depth remains an estimated signal. Severe occlusion, motion blur, reflective surfaces, or illumination outside the training distribution may corrupt both the depth map and its boundaries. The DRGF gate was not validated against ground-truth depth or controlled depth corruption. In addition, although DA3 provides its own model-internal depth-confidence output, the present study did not compare DRGF with a fusion strategy directly weighted by that confidence. Such a comparison would help determine whether task-specific RGB–depth agreement provides information beyond the confidence estimated by the monocular depth model itself. DA3 generation latency was also not measured. Future work should therefore compare native depth-confidence weighting, learned cross-modal gating, and their combination under controlled depth degradation; compare alternative depth estimators; measure the complete RGB-to-detection pipeline on target hardware; and evaluate continuous sequences. Sequence-level testing must determine whether single-frame localization gains reduce identity switches or improve counting and behavior recognition; the current study provides no evidence of those downstream effects.

## 6. Conclusions

DRG-LiteStar-YOLO presents a lightweight, RGB-camera-compatible framework for single-frame dairy goat detection in crowded and unevenly illuminated barns. The method treats monocular pseudo-depth as complementary geometric information rather than a replacement for RGB appearance. Its main contribution is the integration of reliability-guided depth fusion with boundary-enhanced multi-scale feature aggregation, enabling the detector to exploit useful geometric cues while suppressing unreliable depth responses.

On the held-out, unaugmented test partition, the primary seed-42 run achieved a precision of 0.961, a recall of 0.941, an mAP@0.5 of 0.978, and an mAP@0.5:0.95 of 0.735. Compared with the RGB-only LiteStar-YOLO baseline, the proposed model improved mAP@0.5 and mAP@0.5:0.95 by 1.6 and 3.8 percentage points, respectively. The five-seed cumulative ablation maintained a consistent improvement trend, with the full configuration achieving 0.734±0.002 mAP@0.5:0.95 compared with 0.697±0.004 for RGB-only LiteStar-YOLO. These results demonstrate that pseudo-depth provides valuable complementary information when animal boundaries are weakened by occlusion, railings, uneven illumination, and limited RGB contrast.

The proposed detector contains 4.20 M parameters and achieves 86.2 FPS when pseudo-depth maps are precomputed. This combination of compact model size and high detector throughput makes the framework suitable for RGB-camera-based monitoring workflows in which pseudo-depth can be generated offline or cached in advance. The method therefore provides a practical way to improve localization accuracy without introducing a dedicated depth sensor.

The present study establishes the effectiveness of the proposed framework for within-farm dairy goat detection. Future work will extend the evaluation to multiple farms, breeds, cameras, and environmental conditions, while also measuring complete end-to-end latency and validating the method in tracking and other downstream monitoring tasks. Overall, DRG-LiteStar-YOLO offers an effective and deployable approach for improving frame-level goat localization using standard RGB surveillance cameras.

## Figures and Tables

**Table 1:** Description of the dairy goat data acquisition environment.

Item	Description
Farm location	Animal Husbandry Teaching and Experimental Base, Northwest A&F University
Geographical location	Yangling District, Xianyang City, Shaanxi Province, China
Coordinates	108.08° E, 34.27° N
Animal species	Saanen dairy goat
Number of goats	134 lactating dairy goats
Age range	2–5 years
Barn structure	Brick wall and steel roof structure
Indoor stable size	Approximately 25.0 m × 8.0 m × 5.0 m
Outdoor pen size	Approximately 25.0 m × 5.0 m on each side
Total cross-sectional width	Approximately 18.0 m
Outdoor pen wall height	Approximately 1.2 m
Camera model	TP-IPC44CL-V2 fixed RGB camera manufactured by TP-Link Technologies Co., Ltd., Shenzhen, China
Image sensor	1/3-inch CMOS
Lens	4-mm fixed-focus lens
Recording resolution	2560 × 1440 pixels
Video encoding	H.265/HEVC
Mounting geometry	Approximately 2.5 m high with a 30° downward view
Collection period	15 collection days between 15 March and 1 April 2026; approximately 24 h per collection day
Source recordings	248 video files
Frame extraction	Python 3.11.9 and OpenCV 4.13.0.92; 10 f/s; PNG output
Frame selection	64-bit pHash with $d_{H}\leq 6$; maximum Laplacian variance per group with a minimum of 100; manual quality control
Input modality	RGB image and DA3-generated pseudo-depth map
Main visual challenges	Uneven illumination, railings, occlusion, dense distribution, and scale variation
Annotation type	Bounding box annotation for dairy goats

**Table 2:** Key implementation details of DRG-LiteStar-YOLO.

Item	Implementation
Detector input size	640 × 640 pixels
Pseudo-depth estimator	Frozen DA3-Small; offline relative-depth prediction
Detector depth input	Single-channel pseudo-depth map
Depth normalization	Image-wise min–max normalization to [0,1] with $\epsilon =10^{-6}$
Pyramid levels	P3, P4, and P5 with strides 8, 16, and 32
Boundary extraction	Horizontal and vertical Sobel gradients followed by gradient-magnitude calculation
Boundary scales	Boundary map resized to P3, P4, and P5 resolutions
Cross-modal alignment	1×1 convolution
DRGF output	Single-channel sigmoid spatial gate $Q_{l}$
GCA output	Single-channel sigmoid spatial attention map $A_{l}$
Multi-scale fusion	Boundary-enhanced bidirectional feature-pyramid fusion at P3–P5
Training seed (primary comparison)	42
Ablation seeds	0, 21, 42, 3407, 2026
Training precision	Mixed precision (FP16)
Speed-test precision	FP16
Speed-test batch size	1
Speed-test protocol	100 warm-up iterations followed by 1000 timed runs
Speed-test hardware	NVIDIA RTX 4090 GPU
DA3 latency in reported FPS	Excluded; pseudo-depth maps were precomputed
Detailed architecture	Layer-wise definitions and module arguments are provided in the released configuration files and source code

**Table 3:** Source partition and the images used by the detector. No source video contributed images to both training and test; source-video disjointness involving validation was not verified. Augmentation was applied only to the training subset; each training source image contributed its original view and seven photometric variants.

Subset	Source Images	Source Boxes	Images Used	Boxes Used
Training set	1065	9700	8520	77,600
Validation set	304	2830	304	2830
Testing set	152	1455	152	1455
Total	1521	13,985	8976	81,885

**Table 4:** Training settings and hyperparameters.

Item	Setting
Input size	640 × 640
Batch size	16
Training epochs	300
Optimizer	SGD
Initial learning rate	0.01
Minimum recorded learning rate	0.000001
Momentum	0.937
Weight decay	0.0005
Warm-up epochs	3
Random seed (primary comparison)	42
Ablation seeds	0, 21, 42, 3407, 2026
Mixed precision	FP16
Learning rate scheduler	Cosine annealing
Visualization confidence threshold	0.25
NMS IoU threshold	0.45
Deep learning framework	PyTorch 2.1.2 with CUDA 12.1
Hardware platform	NVIDIA RTX 4090 GPU

**Table 5:** Comparison with mainstream object detection models on the dairy goat testing set. Accuracy values are point estimates from the primary seed-42 training run; the cumulative ablation was separately repeated across five seeds. FPS is detector-only; pseudo-depth generation is excluded for DRG-LiteStar-YOLO.

Model	Input	Params (M)	FLOPs (G)	FPS	Precision	Recall	mAP@0.5	mAP@0.5:0.95
Faster R-CNN	RGB	41.30	180.60	18.4	0.902	0.865	0.913	0.621
SSD	RGB	26.30	62.40	46.8	0.869	0.841	0.887	0.552
YOLOv5n	RGB	2.50	7.10	128.6	0.911	0.884	0.932	0.632
YOLOv8n	RGB	3.20	8.70	118.3	0.925	0.897	0.944	0.655
YOLOv10n	RGB	2.70	8.20	125.1	0.928	0.903	0.949	0.666
YOLO11n	RGB	2.60	6.50	132.4	0.932	0.909	0.954	0.675
LiteStar-YOLO	RGB	2.10	5.80	145.7	0.941	0.918	0.962	0.697
DRG-LiteStar-YOLO	RGB + DA3	4.20	12.60	86.2	0.961	0.941	0.978	0.735

**Table 6:** Cumulative ablation study of the proposed modules. Values are mean ± sample SD across five independent training seeds (0, 21, 42, 3407, and 2026). Parameter counts are unchanged across seeds.

Variant	LGD	Depth	DRGF	GCA	DBE- BiFPN	Params (M)	Recall	mAP@ 0.5	mAP@ 0.5:0.95
Baseline YOLO11n	–	–	–	–	–	2.60	0.910±0.003	0.954±0.003	0.676±0.004
LiteStar-YOLO (LGD-Conv)	✓	–	–	–	–	2.10	0.919±0.003	0.962±0.003	0.697±0.004
+DA3 depth branch	✓	✓	–	–	–	3.40	0.926±0.003	0.968±0.003	0.709±0.004
+DRGF	✓	✓	✓	–	–	3.70	0.934±0.003	0.973±0.003	0.724±0.004
+DRGF + GCA	✓	✓	✓	✓	–	3.90	0.938±0.003	0.976±0.003	0.732±0.003
Full model	✓	✓	✓	✓	✓	4.20	0.941±0.003	0.978±0.002	0.734±0.002

**Table 7:** Five-seed robustness of the six cumulative ablation configurations. Values are mean ± SD across seeds 0, 21, 42, 3407, and 2026.

Configuration	Precision	Recall	mAP@0.5	mAP@0.5:0.95
YOLO11n	0.932 ± 0.003	0.910 ± 0.003	0.954 ± 0.003	0.676 ± 0.004
LiteStar + LGD	0.941 ± 0.003	0.919 ± 0.003	0.962 ± 0.003	0.697 ± 0.004
+DA3 depth	0.947 ± 0.002	0.926 ± 0.003	0.968 ± 0.003	0.709 ± 0.004
+DRGF	0.954 ± 0.003	0.934 ± 0.003	0.973 ± 0.003	0.724 ± 0.004
+GCA	0.958 ± 0.003	0.938 ± 0.003	0.976 ± 0.003	0.732 ± 0.003
+DBE-BiFPN	0.961 ± 0.002	0.941 ± 0.003	0.978 ± 0.002	0.734 ± 0.002

**Table 8:** Comparison of input modalities and representative, non-exhaustive multimodal configurations using the same LiteStar backbone. Values are point estimates from the primary seed-42 run; these alternatives were not included in the five-seed cumulative ablation.

Method	RGB	DA3 Depth	Fusion Strategy	Precision	Recall	mAP@ 0.5	mAP@ 0.5:0.95
RGB only	✓	–	–	0.941	0.918	0.962	0.697
Depth only	–	✓	–	0.884	0.852	0.901	0.584
Early fusion	✓	✓	Four-channel input	0.945	0.922	0.965	0.704
Feature concatenation	✓	✓	Direct concatenation	0.949	0.928	0.969	0.715
Attention fusion	✓	✓	Attention-based fusion	0.955	0.934	0.973	0.725
Full DRG-LiteStar-YOLO	✓	✓	DRGF + GCA + DBE-BiFPN	0.961	0.941	0.978	0.735

**Table 9:** Detector -only model complexity and inference efficiency on an RTX 4090 using precomputed pseudo-depth maps. DA3 generation and data-transfer time are excluded and were not measured.

Model	Params (M)	FLOPs (G)	Model Size (MB)	Latency (ms)	FPS
YOLO11n	2.60	6.50	5.40	7.55	132.4
LiteStar-YOLO	2.10	5.80	4.60	6.86	145.7
RGB-depth concat	3.40	10.10	7.20	9.95	100.5
DRG-LiteStar-YOLO	4.20	12.60	8.90	11.60	86.2

## Data Availability

The source code, configuration files, and scripts used for data preparation, pseudo-depth generation, training, evaluation, and visualization are publicly available at https://github.com/yucleon/DRG_LiteStar_YOLO (accessed on 1 September 2026). Instructions and the current download route for the released dataset are provided in the same repository. The public augmentation archive contains 12,168 RGB image files and 111,880 bounding-box annotations. The experiments reported here used 8976 images after source-level partitioning and training-only augmentation, as detailed in Table 3. The repository also documents the required directory structure and preprocessing workflow.
